# Meloxicam and Study of Their Antimicrobial Effects against Phyto- and Human Pathogens

**DOI:** 10.3390/molecules26051480

**Published:** 2021-03-09

**Authors:** Hazem S. Elshafie, Sadeek A. Sadeek, Wael A. Zordok, Amira A. Mohamed

**Affiliations:** 1 School of Agricultural, Forestry, Food and Environmental Sciences, University of Basilicata, Viale dell’Ateneo Lucano 10, 85100 Potenza, Italy; 2 Department of Chemistry, Faculty of Science, Zagazig University, 44519 Zagazig, Egypt; s_sadeek@zu.edu.eg (S.A.S.); wazordok@uqu.edu.sa (W.A.Z.); 3 Department of Chemistry, University College of Quanfudha, Umm Al-Qura University, Quanfudha 21955, KSA, Saudi Arabia; 4 Department of Basic Science, Zagazig Higher Institute of Engineering and Technology, 445519 Zagazig, Egypt; aa.adaim@science.zu.edu.eg

**Keywords:** chelation theory, transition metals, thermal analysis, antimicrobial activity

## Abstract

Recently, the design of new biological metal-ligand complexes has gained a special interest all over the world. In this research, new series of mixed ligand complexes from meloxicam (H_2_mel) and glycine (Gly) were synthesized. Structures of the compounds were investigated employing elemental analyses, infrared, electronic absorption, ^1^H NMR, thermal analyses, effective magnetic moment and conductivity. The estimated molar conductivity of the compounds in 1 × 10^−3^ M DMF solution indicates the non-electrolyte existence of the examined complexes. Additionally, the effective magnetic moment values refer to the complexes found as octahedral molecular geometry. The data of the infrared spectra showed the chelation of H_2_mel and Gly with metal ions from amide oxygen and nitrogen of the thyizol groups of H_2_mel and through nitrogen of the amide group and oxygen of the carboxylic group for Gly. Thermal analyses indicated that the new complexes have good thermal stability and initially lose hydration water molecules followed by coordinated water molecules, Gly and H_2_mel. The kinetic parameters were calculated graphically using Coats–Redfern and Horowitz–Metzeger methods at *n* = 1 and *n* ≠ 1. The density functional theory (DFT) calculations were performed at B3LYP levels. The optimized geometry of the ligand and its complexes were obtained based on the optimized structures. The data indicated that the complexes are soft with η value in the range 0.114 to 0.086, while η = 0.140 for free H_2_mel. The new prepared complexes were investigated as antibacterial and antifungal agents against some phyto- and human pathogens and the minimum inhibitory concentration (MIC) data showed that complex (**A**) has the lowest MIC for *Listeria* and *E. coli* (10.8 µg/mL).

## 1. Introduction

**Scheme 1:**
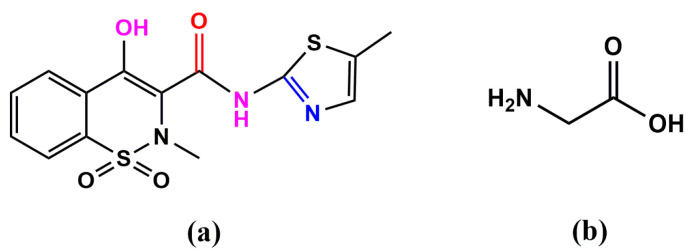
(**a**) H_2_mel (4-hydroxy-2-methyl-*N*-(5-methyl-2-thiazoyl)-2*H*-1,2-benzothiazine-3-carboxamide-1,1-dioxide) and (**b**) glycine (Gly).

Non-steroidal anti-inflammatory drugs (NSAIDs) are utilized to treat irritation, torment, fever and different sorts of cancers such as (colon, stomach, the esophagus, lung, prostate, ovarian and breast cancers) [1,2]. In addition, it can be used efficiently to treat the cardiovascular illness (myocardial localized necrosis, thrombosis and stroke), diabetes (insulin-resistant and the related metabolic disorder) [1,2,3]. NSAIDs reacted with several-metal ions through amide oxygen and nitrogen of the thyizol groups forming complexes, and the literature data revealed that the biological properties of the complexes are more effective than the starting ligands [4,5,6]. The inflammation impact of NSAIDs result from their interaction with the chemical cyclooxygenase (COX), whereas their other natural impacts are COX-independent, counting their impact on tight intersections [1,3]. Meloxicam (H_2_mel) is one of NSAIDs, with the systematic name 4-hydroxy-2-methyl-*N*-(5-methyl-2-thiazolyl)-2*H*-1,2-benzothiazine-3-carboxamide-1,1-dioxide (Scheme 1a). Some metal ions perform a substantial role in biological systems’ medications that may alter the distribution of these ions in the blood plasma and other fluids [4]. The H_2_mel reacted with metal ions forming complexes with octahedral structure and the experimental results refer to meloxicam react as deprotonated bidentate through nitrogen atom of the thiazolyl ring and the amide oxygen with strong intra molecular hydrogen bond between the amide N-H and oxygen atom of enolate [5,6]. Glycine (Gly) (Scheme 1b) is one of the proteinogenic amino acids which is used as buffering agents in food production, antioxidants and reacted with metal ions as a bidentate ligand through lone pair of electrons on nitrogen atom of the amide group and ionic charge on oxygen atom of the carboxylic group in presence of some antimicrobial agents such as moxifloxacin, gemifloxacin and lomefloxacin forming complexes [7,8,9]. Antimicrobial peptides often show broad-spectrum activity due to a mechanism based on bacterial membrane disruption, which also reduces development of permanent resistance, a desirable characteristic in view of the escalating multidrug resistance problem. Peptides with a higher proportion of Gly were generally less potent and caused less bacterial membrane alteration, as observed by flow cytometry and AFM, which correlate to structural characteristics as observed by circular dichroism spectroscopy and predicted by molecular dynamics’ calculations [10,11,12]. It is important to highlight there are limited reports about metal complexes with H_2_mel and Gly [13,14,15,16]. 

In the present work, we synthesized a series of mixed H_2_mel and glycine with some metal ions such as Mn(II), Co(II), Ni(II), Cu(II), Zn(II) and Zr(IV). The complexes were characterized with elemental analysis, diverse spectroscopic techniques (IR, UV-Visible spectra, ^1^H NMR, mass spectra) magnetic susceptibility, molar conductivity and thermogravimetric investigations. The optimized geometry of the ligand and its complexes were obtained based on B3LYP levels using DFT calculations. Biological activity of the innovative compounds was also tested. 

## 2. Results and Discussion

**Scheme 2:**
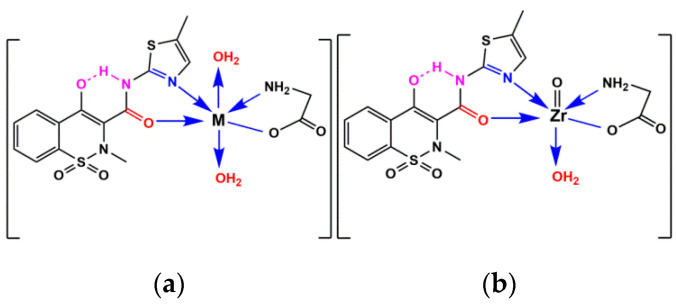
The coordination mode of (**a**) Mn(II), Co(II), Ni(II), Cu(II), Zn(II) and (**b**) Zr(IV) with H_2_mel and Gly.

Metal ions react with the bidentate H_2_mel in presence of Gly forming monomeric complexes. The compounds characterized through elemental analysis, IR, UV-Vis, ^1^H NMR, mass spectroscopy as well as thermal analysis. The data indicate that the metal ions are surrounded by oxygen atom of the amide group and nitrogen atom of the thiazoyl ring for H_2_mel and oxygen atom of carboxylic group with nitrogen atom of the amino group for Gly, and the metal ions complete the coordination number by the oxygen atom of two water molecules in the case of Mn(II), Co(II), Ni(II), Cu(II), Zn(II) and oxygen atom of one water molecule in Zr(IV). The molar conductance measurements in 10^−3^ M DMF of all compounds were found from 8.82 to 26.00 Ω^−1^ mol^−1^ cm^2^ (Supplementary Table S1); the data indicate the compounds found as non-electrolytes [17]. The effective magnetic moments (as B.M.) of the para complexes were measured and indicate the complexes found as octahedral geometries (Scheme 2) [18,19].

### 2.1. Vibrational Spectra 

The infrared spectra of H_2_mel, Gly, (**A**), (**B**), (**C**), (**D**), (**E**) and (**F**) complexes were done (Supplementary Figure S1) and vibrational motion was assigned (Table 1). In the IR spectrum of H_2_mel, ν(N-H)amide, ν(C=O)amide and (C=N) for thiazoyl ring bands appear at 3289, 1620 and 1550 cm^−1^, respectively, while there is an absence of band 3289 cm^−1^ for all prepared compounds, due to the presence of a strong intramolecular hydrogen bond between the N-H group of H_2_mel, also, the shifted of the two peaks for ν(C=O)amide and (C=N) to lower wave number around 1598 and 1520 cm^−1^ which indicate the coordination of the H_2_mel through these two groups [10,20]. Furthermore, the anti-symmetric and symmetric stretching vibrations of the -SO_2_ group in the free H_2_mel found at 1346 and 1183 cm^−1^ are shifted to lower frequencies in the spectra of all complexes. The free Gly exhibit νas(NH2) and νs(NH2) around 3420 and 3169 cm^−1^, respectively. The band observed at 1604 cm^−1^ for free Gly is assigned to the carboxylic group [21,22,23]. In the spectra of the complexes, the difference between asymmetric and symmetric vibration motion of the carboxylic group Δν is found higher than 200 cm^−1^ which indicates that the carboxylate group is chelated in a uni-negative manner to the metal ions [24,25,26,27,28,29,30]. Low intensity bands observed in the range 697–525 cm^−1^ may attribute to ν(M-O) and ν(M-N) stretching vibrations [11,31,32]. Therefore, from the IR spectral studies, it is concluded that H_2_mel and Gly behave as bidentate ligands with NO coordination sites with metal ions.

### 2.2. UV-Visible Spectra

**Figure 1:**
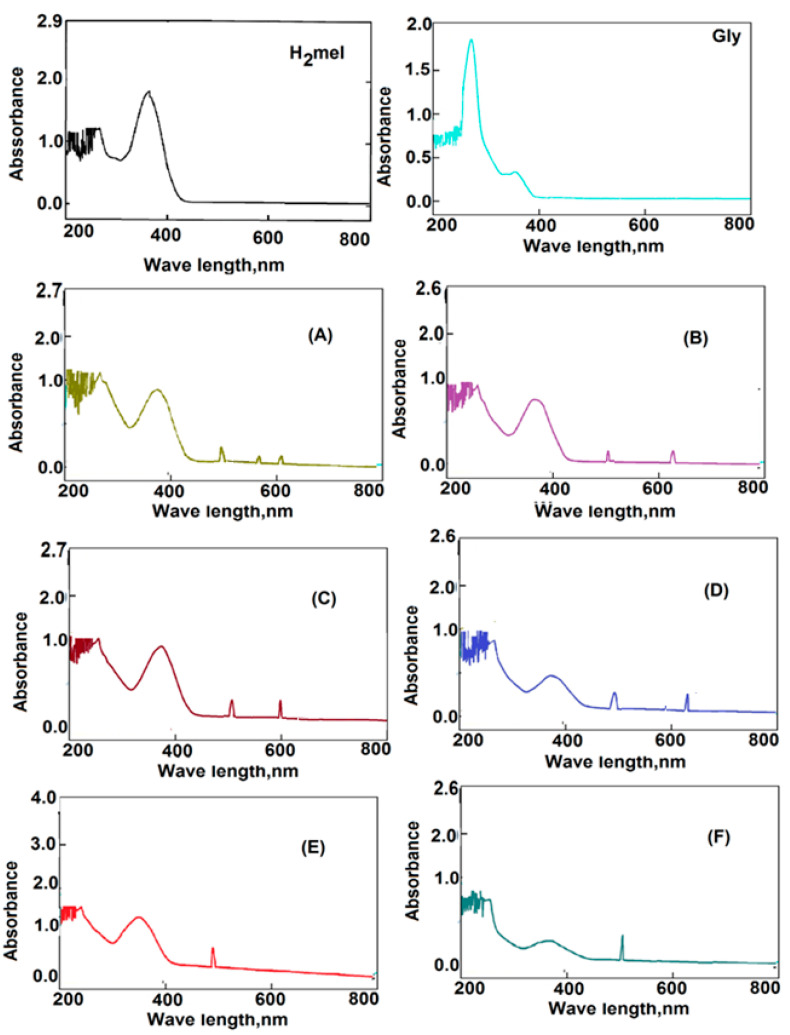
Electronic absorption spectra for H_2_mel, Gly and their metal complexes. Where, (**A**) = Mn(II) complex, (**B**) = Co(II) complex, (**C**) = Ni(II) complex, (**D**) = Cu(II) complex, (**E**) = Zn(II) complex and (**F**) = Zr(IV) complex.

To understand the electronic structure of H_2_mel, Gly, (**A**), (**B**), (**C**), (**D**), (**E**) and (**F**) complexes, the UV-Vis spectra were recorded for these compounds using DMSO as a solvent in the range of 200–800 nm, presented in Supplementary Table S2 and recorded in Figure 1. The absorption of H_2_mel was observed at 266 and 362 nm related to π-π* and n-π*transitions, respectively [12,33,34]. Additionally, Gly absorbed at 290 nm which may assign to n-π*transitions [33,34]. The shift of the absorption bands to higher or lower values and appearance of new bands for the complexes is attributed to chelation of the H_2_mel and Gly with the metal ions. The new bands, in the range 500–528 nm, found in the complexes may be assigned to ligand-metal charge transfer [8,35,36,37,38,39]. The two bands found at 570 and 610 nm in absorbance spectrum of (**A**) complex may be assigned to ^6^A1→^4^T_2_(G) and ^6^A_1g_→^4^T_2g_ (4G) transition, respectively [16] For (**B**) complex, showing absorption band at 605 nm which may be assigned to ^6^T_1g_ (F)→^4^T_1g_ (P) transition, suggest that there is an octahedral geometry around Co(II). For (**C**) complex, the observed band at 600 nm refers to ^3^A_2g_→^3^T_1g_ (P) transition which supports distorted octahedral geometry. The band observed at 625 nm for copper complex may be assigned to ^2^B_1g_→^2^E_g_ transition [8,18]. μ_eff_ value of Cu(II) complex is found at (1.70 B.M.) which was closely related to the spin value (1.73 B.M.) indicating the possibility of an octahedral geometry.

**Figure 2:**
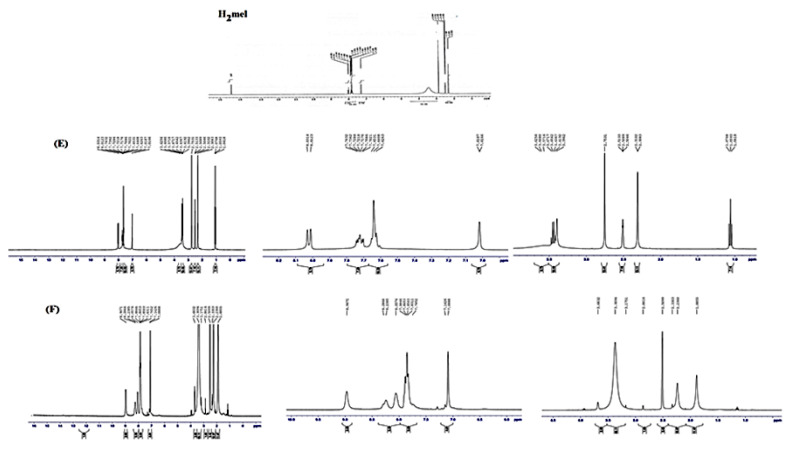
^1^H NMR-DMSO-*d*_6_ spectra for H_2_mel, (**E**) and (**F**) compounds. where (**E**) = Zn(II) complex and (**F**) = Zr(IV) complex.

^1^H NMR spectra of H_2_mel, Gly, (**E**) and (**F**) compounds were done in DMSO-*d*_6_ using a 310 MHz instrument. Due to the resemblance of the aromatic proton signals, they all manifest in a tight range (Figure 2). Spectra of the two complexes showed the aromatic proton signals within the range 7.64–7.88 ppm as overlapped signals as shown in Supplementary Table S3. However, intensity measurements correspond accurately to the total number of aromatic protons in these complexes. An isolated signal was observed at 14.50 ppm and this signal was assigned to the OH enolate in the spectrum of the free H_2_mel, and the disappearing of this peak in the complexes spectra due to H_2_mel is involved in a strong intra molecular hydrogen bond in consistence with the data previously obtained from the infrared spectra. The signal observed at 11.00 ppm assigned to the (-COOH) of Gly molecule disappear in the spectra of all complexes suggesting coordination of Gly through its carboxyl to oxygen atom [40]. The new peaks found in the spectra of (**E**) and (**F**) in the range 3.17–3.68 ppm may be assigned to water molecules and indicated the presence of water molecules in the complexes [41].

### 2.3. Mass Spectra (GC-MS) 

**Scheme 3:**
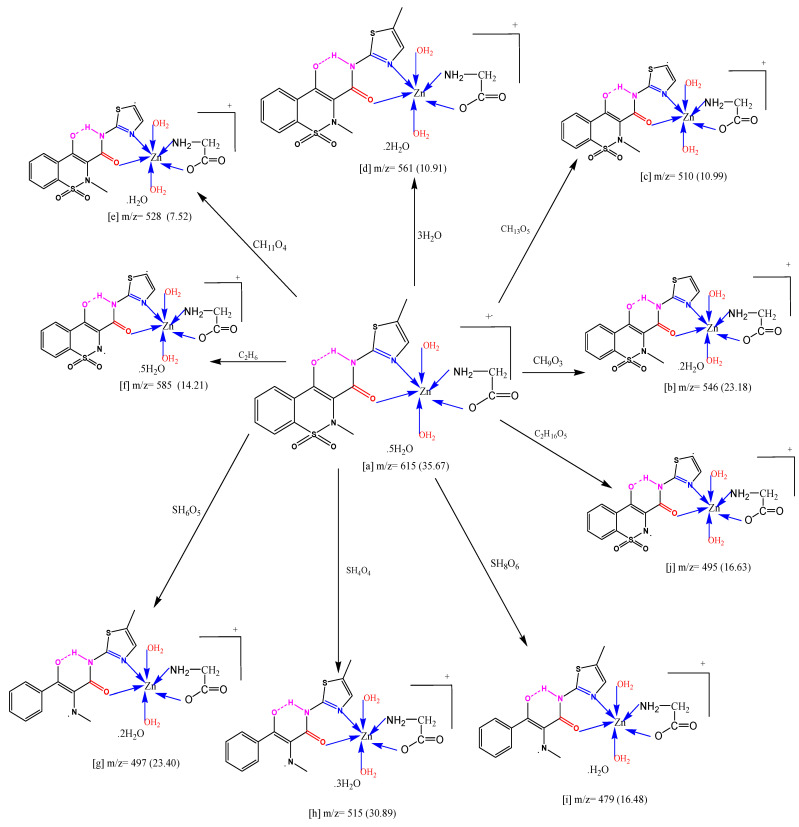
Fragmentation pattern of (**E**) complex.

The mass spectra of complexes confirmed the molecular formulae reported. Mass spectra of all prepared compounds (Supplementary Figure S2) showed that the highest mass peak *m*/*z* = amu at 605 (19.43%), 555 (15.41%), 537 (21.54%), 560 (14.56%), 615 (35.67) and 603 (9.35%), respectively. These data agree very well with the formulae weight of the complexes. Scheme 3 showed the fragmentation pattern of (**E**) complex as representative example. Where the molecular ion peak [a] at *m*/*z* = 615 (35.67%) assigned to loss CH_9_O_3_ to give [b] at *m*/*z* = 546 (23.18%) and loss CH_13_O_5_ to give [c] at *m*/*z* = 510 (10.99%). The molecular ion peak [a] loses 3H_2_O to give [d] at *m*/*z* = 561 (10.91%) and loses CH_11_O_4_ to give [e] at *m*/*z* = 528 (7.52%). The molecular ion peak [a] loses C_2_H_6_ to give fragment [f] at *m*/*z* = 585 (14.21%) and loses SH_6_O_5_ to give fragment [g] at *m*/*z* = 497 (23.40%). The molecular ion peak [a] loses SH_4_O_4_ to give fragment [h] at *m*/*z* = 515 (30.89%) and loses SH_8_O_6_ to give fragment [i] at *m*/*z* = 479 (16.48%). Additionally, (**E**) complex loses the fragment C_2_H_16_O_5_ to give [j] at *m*/*z* = 495 (16.63). 

### 2.4. Thermal Analysis Studies 

Thermal behavior of all compounds was studied by using thermo gravimetric techniques (TGA) at the ambient temperature to 1000 °C. The TGA curves of the compounds are shown in Supplementary Figure S3. The TGA curve of H_2_mel at a maximum temperature 265 °C with 93% weight loss assigned to the loss of 6C_2_H_2_ + 2SO_2_ + 1.5N_2_ + 0.5H_2_ (Table 2). The thermal analysis of Gly is accounted in the literature [42]. The complex (**A**) decomposed in three steps in the temperature range 44–1000 °C. The first step decomposed in the range 44–201 °C with 15.00% weight loss attributed to the liberation of absorbed water molecules (calc. 14.87%). The second step at 298 °C with mass loss 18.00% (calc. 18.18%) may be attributed to the loss coordinated of 2H_2_O and Gly molecules. The third step found at 488 and 773 °C maxima with weight loss 55.14% (calc. 55.24%) corresponding to the loss of H_2_mel molecule with final residue of MnO. Complex (**B**) decomposed within three steps with mass weight loss of 85.63% within the temperature range 44–1000 °C attributed to the loss of lattice water (2H_2_O) in the first step, Gly and coordinated water in the second step and H_2_mel molecule in the third step leaving CoO residue. (**C**) complex decomposed in five steps, with total weight loss 85.38% in the range 43–1000 °C referring to the loss of lattice water molecule, coordinated water molecules, 3C_2_H_4_ + CO_2_ + H_2_S + 1.5N_2_, 5C_2_H_2_ and C_2_H_2_ + C_2_N_2_, respectively, leaving NiO residue. (**D**) complex decomposed in four steps in the range 46–1000 °C with total weight loss of 86.67% corresponding to the loss of C_2_H_4_ + NO_2_ + 4H_2_O, 2C_2_H_2_ + NH_3_ + HCNS, 3C_2_H_2_ and HCN + SO_2_ + CO, respectively. On the other hand, the TGA curve refers the decomposition of the (**E**) complex with two steps. The first one at 109 °C maximum assigned to loss of lattice water molecules. The loss of coordinated water, H_2_mel and Gly was found at 411 and 734 °C maxima with 84.46% (calc. 83.53%) weight loss. The TGA for (**F**) complex shows three decomposition steps. The first one at 100 °C maximum with 8.10% (calc. 8.95%) weight loss assigned to loss of 3H_2_O lattice. The remaining steps were with a 198, 411 and 707 °C maxima with 74.27% (calc. 73.96%) weight loss (Table 2).

The thermodynamic parameters of the compounds at various decomposition stages were calculated and listed in Table 3 using two methods [43,44]. The A and E* values were determined from the intercept and linear slope of each stage (Supplementary Figure S4). Additionally, the parameters, ΔH*, ΔS* and ΔG* were calculated using the equations:

ΔH* = E − RT (1)

ΔS* = R ln (Ah/kBTs)(2)

ΔG* = ΔH* − T ΔS*(3)

where k is the Boltzmann’s constant and h is the Planck’s constant.

The high values obtained for the activation energies (E*) reflect the thermal stability of the complexes. The increasing of ΔG* value for the subsequently decomposition stages indicate that the rate of removal of the subsequent ligand will be lower than that of the precedent ligand and the increasing of TΔS* from one stage to another. This may be attributed to the structural rigidity of the remaining complex after the expulsion of one and more ligands, as compared with the precedent complex, which require more energy, TΔS*, for its rearrangement before undergoing any change. The positive values for ΔS* of the decomposition stages signalized that the disordered structure of activated fragments than of the undecomposed complexes and/or the decomposition reactions are slow [45]. The positive values of ΔH* mean that the decomposition processes are endothermic.

### 2.5. Geometrical Structure of H_2_mel 

**Figure 3:**
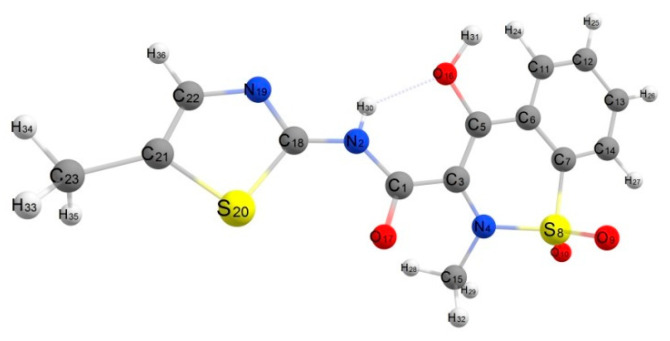
Density functional response theory (DFT) optimized geometry of H_2_mel.

The values of the calculated parameters for optimized geometry of H_2_mel (Figure 3) have some significant results (Supplementary Table S4). (I) The H_2_mel consists of a thiazole and benzothiazene rings joined through by single bond by a carbimide group—CONH, and all of these fragments are lying in the same plane. (II) The formation of hydrogen bond between O16 and H30 (H31-O16···H30-N2) with the bond length is 1.933 Å which is supported by the value of the dihedral angle N2C1C3C5 (31.48°) which indicate the presence of the four atoms lying in the same plane as cis form [46,47]. The dihedral angles O17C1N2C18 and N19C18N2C15 are 59° ≈ 0.0° and 166.87° ≈ 180.0° and proved the O17 and N19 are lying in to two opposite directions in the same plane as trans form. (III) The dihedral angle C1N2C18S20, −8.86° ≈ 0.0° hence the C1=O17 of the –CONH group is lying in the same plane of the thiazole ring and lying in the same direction of S20. (IV) H_2_mel is completely planer and cannot act as bi-dentate ligand O17 of the –CONH group and N19 of the thiazole group without rotation of thiazole group around N2-C18 bond because N19 is lying in trans position respect to O17. (V) In the presence of metal ions, major changes occur in the configuration of H_2_mel molecule [48]. (VI) The above rotation in case of chelation of H_2_mel with metal ions inhibits hydrogen bonding between H30 and O16 as suggested before. The computed data given in Supplementary Table S4 are compatible with those obtained from X-ray data [49]. The charge distribution on H_2_mel is slightly weak dipole, μ = 8.57 D indicates the absence of net positive and net negative poles on the molecule. 

### 2.6. Geometrical Structure of Complexes

MO-treatment has been applied to H_2_mel and complexes in presence of glycine ligand and the equilibrium geometry of each species, metal ion–drug bond formation and charge distribution in studied species were discussed. The structure of the complexes with atomic numbering scheme is shown in Supplementary Figures S5–S10. The complexes consist of one unit of H_2_mel and one unit of Gly beside two water molecules in case of (**A**), (**B**), (**C**), (**D**) and one water molecule for (**F**) forming distorted octahedral bond lengths (Å), bond angles (°), dihedral angles (°), total energy (au), heat of formation (k cal/mol), and dipole moment of the studied complexes were calculated by using DFT calculations (Supplementary Table S5). The data indicated that donating atoms N6 and O9 of Gly and O5 of H_2_mel and O11 are found in equatorial plane while N1 and O10 are found in axial plane. The charge accumulated on donating atoms and metal ions reflects the strong donation from surrounding nitrogen and oxygen atoms of the two mixed ligands.

### 2.7. Charge Distribution Analysis 

The charge distribution analysis on the optimized geometry configuration of H_2_mel and its complexes was made on the basis of natural population analysis (NPA). The charge distribution on H_2_mel refer to the absence of −ve or +ve poles on the molecule; as a result, the H_2_mel is a weak dipole, μ = 8.57 D. The data of charge density reported in Table 4 showed a positive charge density on the metal ion in the (**C**), (**E**) and (**F**) complexes, with the values 0.095, 0.111 and 0.367, respectively, while in case of (**A**), (**B**) and (**D**) complexes, the metal ion carry negative charge density with the value −0.153, −0.052 and −0.117, respectively. The negative charge is delocalized on oxygen, nitrogen and nitrogen atom of carbimide group N3, while all the hydrogen atoms in all complexes carry positive charge. The carbons attached with nitrogen and oxygen atoms (C2, C4, C7 and C9) have more positive values due to electronegative character of oxygen and nitrogen atoms. The results mean that a strong electron donation from donating nitrogen and oxygen atoms of the two mixed ligands to central metal ion in case of (**A**), (**B**) and (**D**), and this strong donation causes generating of a negative charge on these metal ions. In case of (**F**) complex, there is back-donation from the metal sites in a MLCT mode to the π* orbitals of the H_2_mel and Gly. The direction of the dipole moment vector in the complexes which depends on the centers of negative and positive charges is determined according to distribution of atomic charges 

### 2.8. Frontier and Molecular Orbitals 

**Figure 4:**
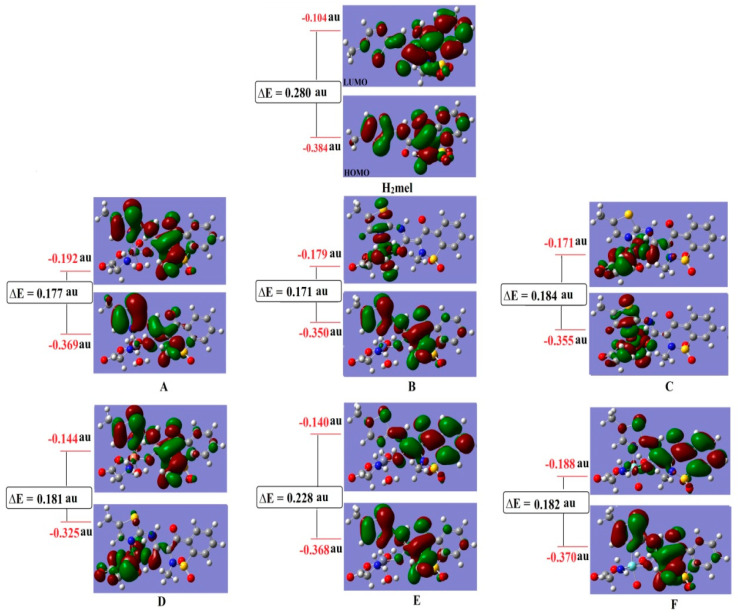
Molecular orbital surfaces and energy levels of H_2_mel and their metal complexes. Where, (**A**) = Mn(II) complex, (**B**) = Co(II) complex, (**C**) = Ni(II) complex, (**D**) = Cu(II) complex, **(E**) = Zn(II) complex and (**F**) = Zr(IV) complex.

Molecular orbitals play an important role in the electric properties, as well as in UV-Vis [50,51]. ΔE (HOMO-LUMO gap) is closely associated with the reactivity and stability of the executed molecule and shows the nature of the molecule with low kinetic stability and slightly high chemical reactivity. The electronic system with smaller values of ΔE may be more reactive than that with greater ΔE [52]. The value of ΔE for the studied complexes varied between 0.171 for (**B**) complex which was more reactive and 0.228 au for (**C**) complex which was less reactive and the electron easily moved between these orbitals with a peak around 250 nm in the UV-Vis spectra for all complexes. On the other hand, the adjacent orbitals are often closely spaced on the frontier region. Figure 4, shows the isodensity surface plots of HOMO and LUMO for free ligand and its complexes, orbital delocalization, strong orbital overlap, and low number of nodal planes. The ΔE value for all complexes depending on the type of metal ion and all complexes have lower ΔE than H_2_mel, (Table 4) which indicate the complexes are more reactive than H_2_mel. For H_2_mel, the electron density of HOMO is delocalized and spreading over all fragments of H_2_mel except C=O of carbimide group and terminal methyl group, while the electron density of LUMO is delocalized and spread over all atoms in H_2_mel except terminal methyl group only. The hardness (η) is defined as (η = (I − A)/2) where I is the ionization energy and A is the electron affinity. 

On the other hand, the (I − A) equals ΔE. Hence, the hardness of H_2_mel and its complexes can be calculated as (η = (EHOMO − ELUMO)/2). Hard molecules have high ΔE, and soft molecules have smaller ΔE. The values of η and ΔE listed in Table 4 indicated that all complexes are soft and η varied from 0.114 for (**E**) complex to 0.086 for (**B**) complex, and 0.140 for H_2_mel. According to the value of ΔE for complexes, the electronic transition within the complexes is easy and these values are in agreement with the values for stable transition metal complexes. The quantum chemical parameters such as global softness (S), electro negativity (χ), absolute softness (σ), chemical potential (Pi), global electrophilicity (ω) and additional electronic charge (ΔN_max_), which depending on ΔE were calculated for the compounds, the (**B**) complex is absolute soft according to the (σ = 11.628 au), while the (**E**) complex is treated as hard complex (σ = 8.772 au). All studied complexes considered as soft with σ varied from 11.628 to 8.772 au, while σ of H_2_mel is 7.14 au. 

### 2.9. Excited State

The TD-DFT at the B3LYP level by using G03W program proved to give accurate description of the UV-Vis spectra [53]. Time-dependent density functional response theory (TD-DFT) has been recently reformulated [54] to compute discrete transition energies and oscillator strengths and has been applied to a number of different atoms and molecules [55]. Bauernschmitt and Ahlrichs [56] included hybrid functional proposed in the calculation of the excitation energies. These hybrid methods typically constitute a considerable improvement over conventional Hartree–Fock (HF)- based methods. In this work, the optimized geometry was calculated and was used in all subsequent calculations; the wave functions of SCF MOs were explicitly analyzed. The calculated wave functions of the different MOs reflect and suggest the fraction of the different fragments of the complex contributing to the total wave functions of different states. 

The results indicate that there is an extent of electron delocalization in the different molecular orbitals. The electronic transition could be described as a mixed n→π* and π→π* transitions. The energies of HOMO and LUMO states for H_2_mel and complexes are listed in Table 4. The HOMO can perform as an electron donor and the LUMO as the electron acceptor in reaction profile. In (**A**), (**B**), (**E**) and (**F**) complexes, the electron density of HOMO is localized mainly on the H_2_mel only, while in case of (**A**), (**E**) and (**F**), the LUMO is localized on all atoms of H_2_mel and central metal ion; in case of (**B**), the LUMO is localized on Gly and terminal end of H_2_mel, so the electronic transition could be described as mixed n→π* and π→π* transitions. The striking feature of the studied (**C**) and (**D**) complexes is that the HOMO and LUMO orbitals are delocalized on the terminal part of H_2_mel and central metal ion with small part on Gly. In (**D**) complex, the HOMO orbital is delocalized and spreading over all surrounding donating atoms in the two ligands around the central metal ion, whereas the LUMO is focused on all atoms of H_2_mel and donating atoms of Gly around Cu(II) ion. In (**C**) complex, the HOMO and LUMO orbitals are localized on the Gly and Ni(II) ion with small portion on the benzothiazine ring.

### 2.10. Antimicrobial Activity 

The antibacterial and antifungal activity of our compounds were screened against *E. coli*, *Coliform*, *S. aureus*, *S. typhi*, *Citrobacter*, *Listeria* and *A. niger* compared to amoxycillin/clavulanic and cetaxime (antibacterial agents). The obtained data are listed in Table 5. In order to increase the chance of detecting antibiotic principles, more than one tested run was used. The (**E**) complex showed high significance against *Listeria*, *E. coli* and *Coliform*. The (**C**) complex showed highly significance against *Listeria* and non-significance against *Coliform* and *E. coli*, whereas (**F**) complex showed very high significance against *E. coli*. H_2_mel showed high significance against *E. coli* and *Citrobacter*. The results ensured that Gly, (**A**), (**B**) and (**D**) compounds were more active than all other compounds; on the other hand, none of the tested compounds explicated antifungal activity. Moreover, all the precursor metal salts did not show antibacterial and antifungal activities. The marked activity of the metal complexes may be explained according to the chelation process which may facilitate the ability of a complex to cross a cell membrane and facilitate their diffusion through the lipid layer of spore membrane to the site of action [57,58]. The variation in the activity of the metal complexes against different organisms depends on their permeability of each metal ion. The minimum inhibitory concentration (MIC; µg) for H_2_mel, Gly, (**A**), (**B**), (**C**), (**D**), (**E**) and (**F**) compounds were evaluated and showed that the complex (**A**) has the lowest MIC for *Listeria* and *E. coli* (10.8 µg/mL); also, the lowest MIC for Gly is (12.8 µg/mL) against *Coliform*.

## 3. Materials and Methods

### 3.1. Materials 

All used chemical reagents: manganese(II) chloride dihydrate MnC_l2_·H_2_O, cobalt(II) chloride hexahydrate CoC_l2_·6H_2_O, nickel(II) acetate dihydrate Ni(CH_3_COO)_2_·2H_2_O, copper(II) chloride dihydrate CuC_l2_·2H_2_O, zinc(II) chloride hydrate ZnCl_2_·H_2_O, zirconium(IV) oxychloride octahydrate ZrOC_l2_·8H_2_O, AgNO_3_, glycine (Gly), KOH and organic solvents ethanol, *N*,*N*-dimethyl formamide (DMF), dimethyl sulfoxide (DMSO) and (DMSO-*d*_6_) were purchased from Fluka, Aldrich Chemical Co. (Cairo, Egypt) and Amoun Pharmaceutical Company (El-Obour, Egypt).

### 3.2. Preparation of Metal-Ligand Complexes 

The metal-ligand complexes were prepared according to the following procedure. The dark yellow [Mn(Hmel)(Gly)(H_2_O)_2_]·5H_2_O solid complex (**A**) was prepared by adding 1 mmol (0.143 g) of MnCl_2_·H_2_O in 20 mL ethanol to mixture from 1 mmol (0.351 g) of (H_2_mel), 1 mmol (0.0750 g) of Gly in presence of 2.0 mmol (0.110 g) of KOH in 40 mL absolute ethanol. The mixture was refluxed for 3 h and the formed precipitate filtered off and dried under vacuum over anhydrous CaCl_2_. The orange, pale green, dark green, pale yellow, and yellowish complexes [Co(Hmel)(Gly)(H_2_O)_2_]·2H_2_O (**B**), [Ni(Hmel)(Gly)(H_2_O)_2_]·H_2_O (**C**), [Cu(Hmel)(Gly)(H_2_O)_2_]·2H_2_O (**D**), [Zn(Hmel)(Gly)(H_2_O)_2_]·5H_2_O (**E**) and [ZrO(Hmel)(Gly)]·3H_2_O (**F**) were prepared as described above using CoCl_2_·6H_2_O, Ni(CH_3_COO)_2_·2H_2_O, CuCl_2_·2H_2_O, ZnCl_2_·H_2_O and ZrOCl_2_·8H_2_O, respectively, in ethanol as a solvent with 1:1:1:2 (Mn +:H_2_mel:Gly:KOH) molar ratio, with the yield range from 78.2 to 89.5%.

### 3.3. Computational Studies

The equilibrium structure of H_2_mel and its complexes were evaluated by density functional theory using GAUSSIAN 98W programs (Pittsburg, CA, USA) and B3LYP/CEP-31G level of theory [59,60]. The atomic charges were computed using the natural atomic orbital populations. The high basis set was chosen to detect the energies at a highly accurate level. 

### 3.4. Instruments 

The elemental analyses were performed using a Perkin Elmer 2400CHN elemental analyzer (Cairo University, Giza, Egypt). The percentage of the metal ions was determined using atomic absorption method. Spectrometer model PYE-UNICAM SP 1900 (Cairo University, Giza, Egypt) fitted with the corresponding lamp was used; also, the percent of the metal ions determined gravimetrically by transforming the metal solid complexes into metal oxide or metal. Infrared spectra (4000–400 cm^−1^) were recorded as KBr discs with FTIR 460 PLUS Spectrophotometer (Cairo University, Giza, Egypt). Electronic spectra were recorded on UV-3101PC Shimadzu (Cairo University, Giza, Egypt). ^1^H NMR spectra were recorded using Varian Mercury VX-300 NMR Spectrometer (Cairo University, Giza, Egypt). Dimethyl sulfoxide (DMSO-*d*_6_) was used as a solvent and tetramethylsilane (TMS) as an internal reference. Mass spectra in the range from 0–1090 were recorded on GCMS-QP-1000EX Shimadzu (ESI-70ev) (Al-Azhar University, Cairo, Egypt). Effective magnetic moments were done on a Sherwood scientific magnetic balance (Cairo University, Giza, Egypt) using Gouy balance at room temperature. Molar conductivities of 10^−3^ M solutions of solid compounds in DMF were measured on the CONSORT K410 (Zagazig University, Zagazig, Egypt). Melting points were measured on a Buchi apparatus. TGA/DTG measurements were carried out from ambient temperature up to 1000 °C at a heating rate of 10 °C/min under nitrogen atmosphere on a TGA-50H Shimadzu (Cairo University, Giza, Egypt).

### 3.5. Antimicrobial Investigation

The antimicrobial activity of the H_2_mel, Gly and their complexes was carried out following the disc diffusion method as described by Elshafie et al. [41,61] against the following human and phytopathogens: *Escherichia coli* ATCC11229, *Coliform* ATCC8729, *Staphylococcus aureus* ATCC6538, *Salmonella typhi* ATCC14028, *Citrobacter*, Listeria and Aspergillus niger. The nutrient agar medium for antibacterial test was prepared as following: 0.5% peptone, 0.1% beef extract, 0.2% yeast extract, 0.5% NaCl and 1.5% agar-agar), whereas Czapeks-Dox medium for antifungal test was prepared as following: 3% sucrose, 0.3% NaNO_3_, 0.1% K_2_HPO_4_, 0.05% KCl, 0.001% FeSO_4_ and 2% agar-agar. The two prepared media were autoclaved at 121 °C for 20 min, then were cooled to 45 °C and inoculated with each tested microorganisms. Each nutrient media was poured, about 14 mL, in Petri dishes of 90 mm diameter. After solidification, 5 mm diameter holes were punched by a sterile cork-borer, and 0.1 mL from each tested compound, dissolved in DMF at 1.0 × 10^−3^ was applied in each hole. All plates were incubated for 24 h at 37 °C bacteria and for 96 h at 24 °C for fungi. The antimicrobial activity was determined by measuring the diameter of the inhibition zone (in mm). 

### 3.6. Minimum Inhibitory Concentration (96-Well Microplate Method)

The minimum inhibitory concentration (MIC) has been carried out against all tested pathogens using 96-well microplates (Nunc MaxiSorp^®^, Vedbaek, Denmark) by a micro-dilution method, as reported by Elshafie et al. [9]. A 4 mL liquid suspension from fresh microbial cultures was prepared at 10^6^ CFU/mL for bacteria and 10^8^ spore/mL for fungi. Two hundred µL/well from each tested compounds were dissolved in broth king B media (KB) for bacteria and potato dextrose broth (PDB) for fungi at the concentrations ranging from 0 to 100 µg/mL; with interval of 10 µg/mL and 100 µL/well of each microbial suspension added in the microplate and then incubated at 37 ± 2 °C for bacteria and 22 ± 2 °C for fungi. The absorbance was measured at λ = 450 nm using an Elisa microplate reader instrument (DAS s.r.l., Rome, Italy) after 24 h for bacteria and 72 h for fungi. The whole experiment was repeated in triplicate. The minimum inhibitory concentration of the antimicrobial activity was presented as µg/mL compared to the positive control.

## 4. Conclusions

The depiction and installation of the novel new series of mixed ligand complexes from H_2_mel and Gly with Mn(II), Co(II), Ni(II), Cu(II), Zn(II) and Zr(IV) were synthesized. The knowledge centered on the data outcomes from physico-chemical and spectral technique showed that H_2_mel and Gly behaves as bidentate with metal ions via NO sites. FT-IR and TGA data confirmed the presence of water molecules inside and outside the complex sphere. Magnetic susceptibility data suggests the complexes of Mn(II), Co(II), Ni(II) and Cu(II) were found as octahedral geometry and the molar conductivity data indicated non-electrolyte existence for all complexes. Kinetic parameters (activation entropy, activation energy, activation enthalpy, and Gibbs free energy at *n* = 1 and *n* ≠ 1) were calculated. The measurements of the molecular modeling using density functional theory confirm the structural geometry of the complexes is soft with respect to the ligands. The new prepared complexes were investigated as antibacterial and antifungal agents against some phyto- and human pathogens and the minimum inhibitory concentration data showed that complex (A) has 10.8 µg/mL in case of Listeria and *E. coli*.

## Figures and Tables

**Table 1:** Selected infrared absorption frequencies (cm^−1^) for H_2_mel, Gly and their metal complexes.

Compounds	ν(O-H); Enolate; H_2_O	ν(N-H)	ν(NH_2_)	ν(C=O); Amide	ν(C=O) COOH	ν_as_(COO^−^)	ν(C=N) Thiazoyl Ring	ν_s_(COO^−^)	ν_as_(so_2_)	ν_s_(so_2_)	ν(Zr=O)	ν(M-O) and ν(M-N)
H_2_mel	3438 br	3289 s,sh		1620 s	-	----	1550 vs		1346 s	1183 s	----	----
Gly	-----	-----	3420 br 3169 m		1604 vs	---	-----				-----	----
(A)	3459 m	------	3376 w 3165 m	1608 vs	-------	1597 m	1515 m	1394 m	1327 s	1168 vs	---	652 m, 610 m, 574 m and 530 m
(B)	3403 m	------	3380 vw 3100 vw	1614 vs	--------	1599 m	1514 s	1398 vs	1331 vs	1165 s	----	671 w, 623 w and 576 m
(C)	3395 m, br	----	3256 w 3105 vw	1606 vs	-------	1599 m	1510 s	1398 s	1335 s	1174 s	-----	628 m, 573 m and 526 vw
(D)	3449 w	-----	3265 w 3106 vw	1593 vs	----	1590 m	1509 m	1389 s	1336 s	1167 s	----	664 w, 625 m, 574 m and 529 w
(E)	3400 w	-----	3379 w 3182 m	1610 vs	----	1597 m	1511 s	1396 s	1334 s	1175 s	------	670 m, 624 m, 571 m and 526 m
(F)	3422 s	----	3200 w 3100 w	1612 vs	-----	1568 sh	1523 m	1365 w	1348 s	1171 s	856 m	697 m, 659 m, 579 m and 525 m

Key: s = strong, w = weak, m = medium, br = broad, ν = stretching.

**Table 2:** The maximum temperature T_max_ (°C) and weight loss values of the decomposition stages for H_2_mel, Gly and their metal complexes.

Lost Species	Weight Loss (%)		T_max_ (°C)	Decomposition	Compounds
	Found	Calc.			
6C_2_H_2_ + 2SO_2_ + 1.5N_2_ + 0.5H_2_	93.00	93.10	265	First step	H_2_mel
					(C_14_H_13_N_3_O_4_S_2_)
	93.00	93.10		Total loss	
2C	7.00	6.80		Residue	
CO + NH_3_ + H_2_O	83.38	84.01	255	First step	Gly
	83.38	84.01		Total loss	(C_2_H_5_NO_2_)
C	16.62	15.99		Residue	
5H_2_O	15.00	14.87	136	First step	(**A**)
C_2_H_4_ + NO_2_ + 2H_2_O	18.00	18.18	298	Second step	(MnC_16_H_30_N_4_O_13_S_2_)
6C_2_H_2_ + C_2_N_2_ + NO + 2SO	55.14	55.24	488, 773	Third step	
	88.14	88.29		Total loss	
MnO	11.86	11.71		Residue	
2H_2_O	7.00	6.49	105	First step	(**B**)
C_2_H_4_ + NO_2_ + 2H_2_O	20.00	19.82	374	Second step	(CoC_16_H_24_N_4_O_10_S_2_)
6C_2_H_2_ + C_2_N_2_ + NO + 2SO	58.63	60.20	553, 798	Third step	
	85.63	86.51		Total loss	
CoO	14.37	13.49		Residue	
H_2_O	3.30	3.35	100	First step	(**C**)
2H_2_O	6.74	6.71	198	Second step	(NiC_16_H_22_N_4_O9S_2_)
3C_2_H_4_ + CO_2_ + H_2_S + 1.5N_2_	37.00	37.24	309	Third step	
5C_2_H_2_	24.17	24.20	561	Fourth step	
C_2_H_2_ + C_2_N_2_	14.17	14.60	814	Fifth step	
	85.38	86.10		Total loss	
NiO	14.62	13.90		Residue	
4H_2_O + C_2_H_4_ + NO_2_	26.10	26.09	185	First step	(**D**)
2C_2_H_2_ + NH_3_ + HCNS	23.00	22.86	366	Second step	(CuC_16_H_24_N_4_O_10_S_2_)
3C_2_H_2_	13.82	13.92	552	Third step	
HCN + SO_2_ + CO	23.75	23.64	790	Fourth step	
	86.67	86.51		Total loss	
Cu + C	13.33	13.49		Residue	
2H_2_O	5.30	5.85	109	First step	(**E**)
6C_2_H_2_ + C_2_N_2_ + C_2_H_4_ + 2NO_2_ + 2SO + 5H_2_O	84.46	83.53	411, 734	Second step	(ZnC_16_H_30_N_4_O_13_S_2_)
	89.76	89.38		Total loss	
Zn	10.24	10.62		Residue	
3H_2_O	8.10	8.95	100	First step	(**F**)
C_2_H_4_ + NO_2_ + H_2_O	15.00	15.25	198	Second step	(ZrC_16_H_24_N_4_O_11_S_2_)
6C_2_H_2_ + CO + 2SO + NO_2_ + N_2_	59.27	58.71	411, 707	Third step	
	82.38	82.91		Total loss	
Zr + C	17.62	17.09		Residue	

**Table 3:** Thermal behavior and kinetic parameters determined using Coats–Redfern (CR) and Horowitz–Metzger (HM) operated for H_2_mel, Gly and their metal complexes.

Compounds	Decomposition Range (K)	T_s_ (K)	Method	Parameters					R ^a^	SD ^b^
				E* (KJ/mol)	A (s^−1^)	ΔS* (KJ/mol·K)	ΔH* (KJ/mol)	ΔG* (KJ/mol)		
H_2_mel (C_14_H_13_N_3_O_4_S_2_)	467–1273	538	CR	71.39	3.99 × 10^3^	−0.1809	66.92	164.28	0.967	0.324
			HM	55.49	9.35 × 10^2^	−0.1929	51.01	154.82	0.953	0.386
Gly (C_2_H_5_NO_2_)	468–588	528	CR	68.88	1.41 × 10^4^	−0.1701	64.49	154.34	0.980	0.223
			HM	65.80	1.52 × 10^4^	−0.1695	61.41	150.94	0.971	0.271
(**A**) (MnC_16_H_30_N_4_O_13_S_2_)	669–826	731	CR	362.765	2.09 × 10^23^	0.1940	356.68	214.80	0.974	0.287
			HM	348.03	9.65 × 10^22^	0.1876	341.96	204.79	0.972	0.303
	826–1273	1046	CR	406.68	9.11 × 10^17^	0.0884	397.98	305.44	0.995	0.084
			HM	401.42	8.16 × 10^17^	0.0875	392.73	301.14	0.995	0.087
(**B**) (Co C_16_H_24_N_4_O_10_S_2_)	464–748	647	CR	96.26	2.08 × 10^6^	−0.13038	90.88	175.24	0.962	0.335
			HM	126.54	9.94 × 10^7^	−0.0982	121.16	184.73	0.950	0.377
	948–1273	1071	CR	359.69	3.58 × 10^14^	0.0230	27.06	23.40	0.977	0.220
			HM	421.60	2.87 × 10^2^	0.1552	412.70	246.41	0.977	0.236
(**C**) (Ni C_16_H_22_N_4_O_9_S_2_)	514–765	583	CR	131.42	1.37 × 10^8^	−0.0946	126.58	181.76	0.961	0.336
			HM	88.55	4.47 × 10^5^	−0.1423	83.70	166.67	0.958	0.363
	766–944	834	CR	349.18	6.30 × 10^22^	0.1830	342.24	189.61	0.970	0.308
			HM	358.42	2.89 × 10^20^	0.1382	351.48	236.18	0.960	0.340
	944–1273	1087	CR	983.30	1.07 × 10^46^	0.6255	974.26	294.31	0.975	0.279
			HM	101.71	1.30 × 10^47^	0.6462	1008.09	305.59	0.973	0.287
(**D**) (Cu C_16_H_24_N_4_O_10_S_2_)	558–750	639	CR	172.48	2.74 × 10^11^	−0.0322	167.16	187.78	0.985	0.194
			HM	162.47	1.52 × 10^11^	−0.0371	157.16	180.92	0.981	0.215
	751–949	825	CR	694.12	2.58 × 10^43^	0.5777	687.26	210.65	0.995	0.995 0.100
			HM	736.991	9.97 × 10^44^	0.6080	730.13	228.46	0.995	
	950–1273	1063	CR	412.43	9.23 × 10^17^	0.0884	403.59	309.57	0.977	0.221
			HM	415.33	1.88 × 10^18^	0.0943	406.49	306.16	0.974	0.236
(**E**) (Zn C_16_H_30_N_4_O_13_S_2_))	426–827	684	CR	35.79	15.30	−0.2291	30.11	186.83	0.987	0.176
			HM	83.31	8.20 × 10^3^	−0.1768	77.63	198.62	0.987	0.176
	827–1273	1007	CR	756.40	3.9 × 10^36^	0.4455	748.03	299.36	0.956	0.312
			HM	745.78	7.1 × 10^36^	0.4504	737.41	283.77	0.953	0.329
(**F**) (ZrC_16_H_26_N_4_O_10_S_2_)	414–552	471	CR	120.00	1.42 × 10^11^	−0.0351	116.09	132.65	0.988	0.163
			HM	120.08	2.24 × 10^11^	−0.0313	116.17	130.95	0.984	0.185
	553–847	684	CR	279.65	2.88 × 10^14^	0.0249	273.95	256.83	0.986	0.181
			HM	174.96	1.63 × 10^13^	0.0011	169.27	168.48	0.984	0.297
	848–1267	980	CR	229.81	3.53 × 10^17^	0.0811	221.66	142.14	0.971	0.297
			HM	600.29	1.24 × 10^30^	0.3213	592.14	277.27	0.967	0.317

a = correlation coefficients of the Arrhenius plots and b = standard deviation. (*) is the activation energy.

**Table 4:** Calculated charges on donating sites and energy values (HOMO, LUMO, energy gap ΔE/eV, hardness (η), global softness (S), electro negativity (χ), absolute softness (σ), chemical potential (Pi), global electrophilicity (ω) and additional electronic charge (ΔN_max_) of the H_2_mel and studied complexes by using DFT calculations.

Parameters	H_2_mel	(A)	(B)	(C)	(D)	(E)	(F)
M	-	−0.153	−0.052	0.095	−0.117	0.111	0.367
N_thiazole_	−0.318	−0.174	−0.139	−0.153	−0.133	−0.147	−0.158
O_carbimide_	−0.592	−0.418	−0.416	−0.481	−0.384	−0.461	−0.387
N6_Gly_	-	−0.172	−0.178	−0.185	−0.179	−0.185	−0.173
O9_Gly_	-	−0.444	−0.438	−0.513	−0.393	−0.495	−0.408
HOMO, H	−0.384	−0.369	−0.350	−0.355	−0.325	−0.368	−0.370
LUMO, L	−0.104	−0.192	−0.179	−0.171	−0.144	−0.140	−0.188
I = −H	0.384	0.369	0.350	0.355	0.325	0.368	0.370
A = −L	0.104	0.192	0.179	0.171	0.144	0.140	0.188
ΔE = L − H	0.28	0.177	0.171	0.184	0.181	0.228	0.182
η = (I − A)/2	0.140	0.089	0.086	0.092	0.091	0.114	0.091
χ = −(H − L/2)	0.244	0.281	0.265	0.263	0.235	0.254	0.279
σ = 1/η	7.14	11.236	11.628	10.869	10.989	8.772	10.989
S = 1/2 η	3.57	5.618	5.814	5.435	5.495	4.386	5.495
Pi = −χ	−0.244	−0.281	−0.265	−0.263	−0.235	−0.254	−0.279
ω = (Pi)2/2 η	0.213	0.444	0.775	0.376	0.303	0.283	0.428
ΔN_max_ = χ/η	1.74	3.16	3.081	2.859	2.582	2.228	3.066

(I) is ionization energy and (A) is an electron affinity.

**Table 5:** The inhibition diameter zone (mm) and minimum inhibition concentration (µg/mL) values for H_2_mel, Gly and their metal complexes.

Tested Compounds	Tested Microorganism													
	Bacteria												Fungi	
	*S. aureus*	MIC	*Listeria*	MIC	*E. coli*	MIC	*Coliform*	MIC	*Citrobacter*	MIC	*S. typhimurium*	MIC	*A. niger.*	MIC
H_2_mel	0	-	0	-	7.3^+2^ ± 0.8	50.0 ± 0.02	0	-	4.6^+3^ ± 0.3	30.0 ± 0.1	0	-	0	
Gly	0	-	5^+2^ ± 0.8	12.8 ± 1.3	4^+2^ ± 0.9	25.6 ± 2.3	6^+2^ ± 1.2	12.8 ± 1.2	0	-	0	-	0	-
(**A**)	0	-	4^+2^ ± 0.5	10.8 ± 1.1	2^+2^ ± 0.2	10.8 ± 1.2	6^+2^ ± 1.1	21.6 ± 1.8	0	-	0	-	0	-
(**B**)	0	-	3^+2^ ± 0.2	44.32 ± 2.3	7^+2^ ± 1.1	22.16 ± 1.5	7^+2^ ± 1.3	22.16 ± 1.6	0	-	0	-	0	-
(**C**)	0	-	1 ± 0.1^+2^	32.16 ± 2.1	0	-	0	-	0	-	0	-	0	-
(**D**)	0	-	2^+2^ ± 0.4	20.96 ± 1.8	5^+2^ ± 0.9	20.96 ± 1.9	7^+2^ ± 1.2	20.96 ± 2.1	0	-	0	-	0	-
(**E**)	0	-	2^+2^ ± 0.2	24.632 ± 1.2	6^+2^ ± 1.1	36.94 ± 2.3	0	-	0	-	0	-	0	-
(**F**)	0	-	0	-	2^+3^ ± 0.1	36.21 ± 2.5	0	-	0	-	0	-	0	
Control (DMF)	0.26 ± 0.012		0.14 ± 0.01		0.26 ± 0.02		0.35 ± 0.022		0.35 ± 0.022		0	-	0	
A/C	1.9 ± 0.4		1 ± 0.1		1.7 ± 0.2		1.4 ± 0.3		0		0		0	
Cetaxime	0	-	0	-	0	-	0	-	0	-	0	-	0	-

Statistical significance P^NS^ P not significant, *p* > 0.05; P^+1^ P significant, *p* < 0.05; P^+2^ P highly significant, *p* < 0.01; P^+3^ P very highly significant, *p* < 0.001; student’s *t*-test (paired). MIC: minimum inhibition concentration (µg/mL); DMF: dimethyl formamide; A/C = amoxycillin/clavulanic.

## Data Availability

The data presented in this study are available on request from the corresponding author.

## Supplementary Materials

The following are available online, Figure S1: Infrared spectra for H_2_mel, Gly and their metal complexes, Figure S2: Mass spectra diagrams for H_2_mel, Gly and their metal complexes, Figure S3: TG diagram for H_2_mel, Gly and their metal complexes, Figure S4: The diagrams of kinetic parameters of H_2_mel, Gly and their metal complexes using Coats–Redfern (CR) and Horowitz–Metzger (HM) equations, Figure S5: DFT-optimized geometry of (A) complex, Figure S6: DFT-optimized geometry of (B) complex, Figure S7: DFT-optimized geometry of (C) complex, Figure S8: DFT-optimized geometry of (D) complex, Figure S9: DFT-optimized geometry of (A) complex, Figure S10: DFT-optimized geometry of (A) complex. Table S1: Elemental analysis and physico-analytical data for H_2_mel, Gly and their metal complexes, Table S2: UV-Vis spectra for H_2_mel, Gly and their metal complexes, Table S3: ^1^H NMR values (ppm) and tentative assignments for H_2_mel, Gly and their metal complexes. Table S4: Equilibrium geometric parameters’ bond lengths (Å), bond angles (°), dihedral angles (°), total energy (eV), heat of formation (k cal/mol) and dipole moment of the H_2_mel by using DFT calculations. Table S5: Equilibrium geometric parameters’ bond lengths (Å), bond angles (°), dihedral angles (°), total energy (eV), heat of formation (k cal/mol) and dipole moment of the studied complexes by using DFT calculations.

## Sample Availability

Samples of the compounds are available from the corresponding author.
