# Supplementary material for: Meloxicam and Study of Their Antimicrobial Effects against Phyto- and Human Pathogens
Source: Molecules. 2021 Mar 9;26(5):1480. doi: 10.3390/molecules26051480 (PMC7963159; doi:10.3390/molecules26051480)
Supplement: Supplementary file 1 [file molecules-26-01480-s001.pdf]

Meloxicam and Study of Their Antimicrobial Effects Against Phyto- and Human Pathogens

Hazem S. Elshafie[1,*] Amira A. Mohamed[2], Sadeek A. Sadeek[3], Wael A. Zordok[3,4]

[1] School of Agricultural, Forestry, Food and Environmental Sciences, University of Basilicata, Viale dell'Ateneo Lucano 10, 85100 Potenza, Italy; hazem.elshafie@unibas.it (H.S.E.);

[2] Department of Basic Science, Zagazig Higher Institute of Engineering and Technology, Zagazig, Egypt; aa.adaim@science.zu.edu.eg (A.A.M.)

[3] Department of Chemistry, Faculty of Science, Zagazig University, 44519 Zagazig, Egypt; s_sadeek@zu.edu.eg (S.A.S.)

[4] Department of Chemistry, University College of Quanfudha, Umm Al -Qura University, KSA, Saudi Arabia; wazordok@uqu.edu.sa (W.A.Z.);

* Correspondence: hazem.elshafie@unibas.it; Tel.: +39-0971-205522; Fax: +39-0971-205503

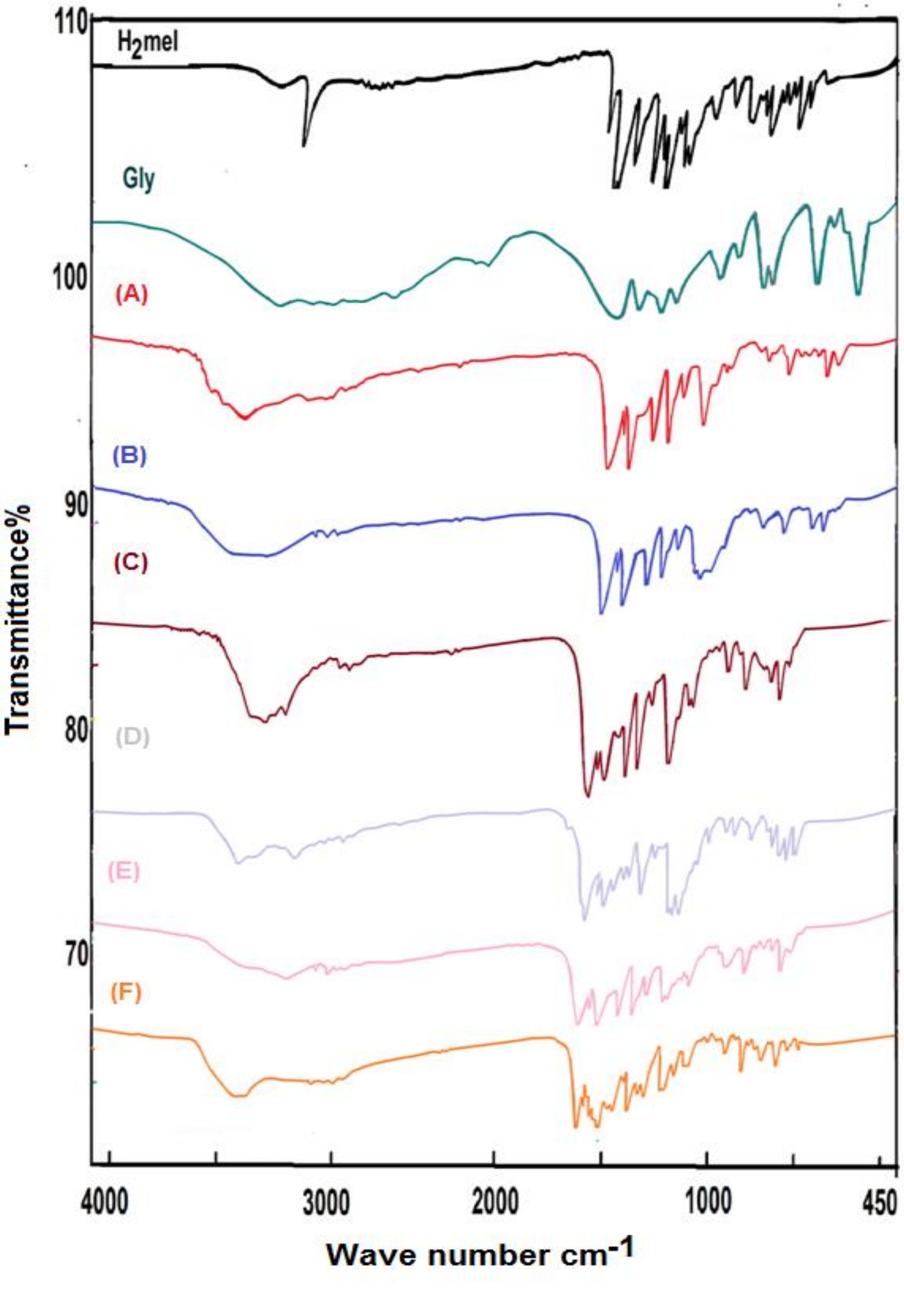

Figure S1 Infrared spectra for H_2mel, Gly and their metal complexes

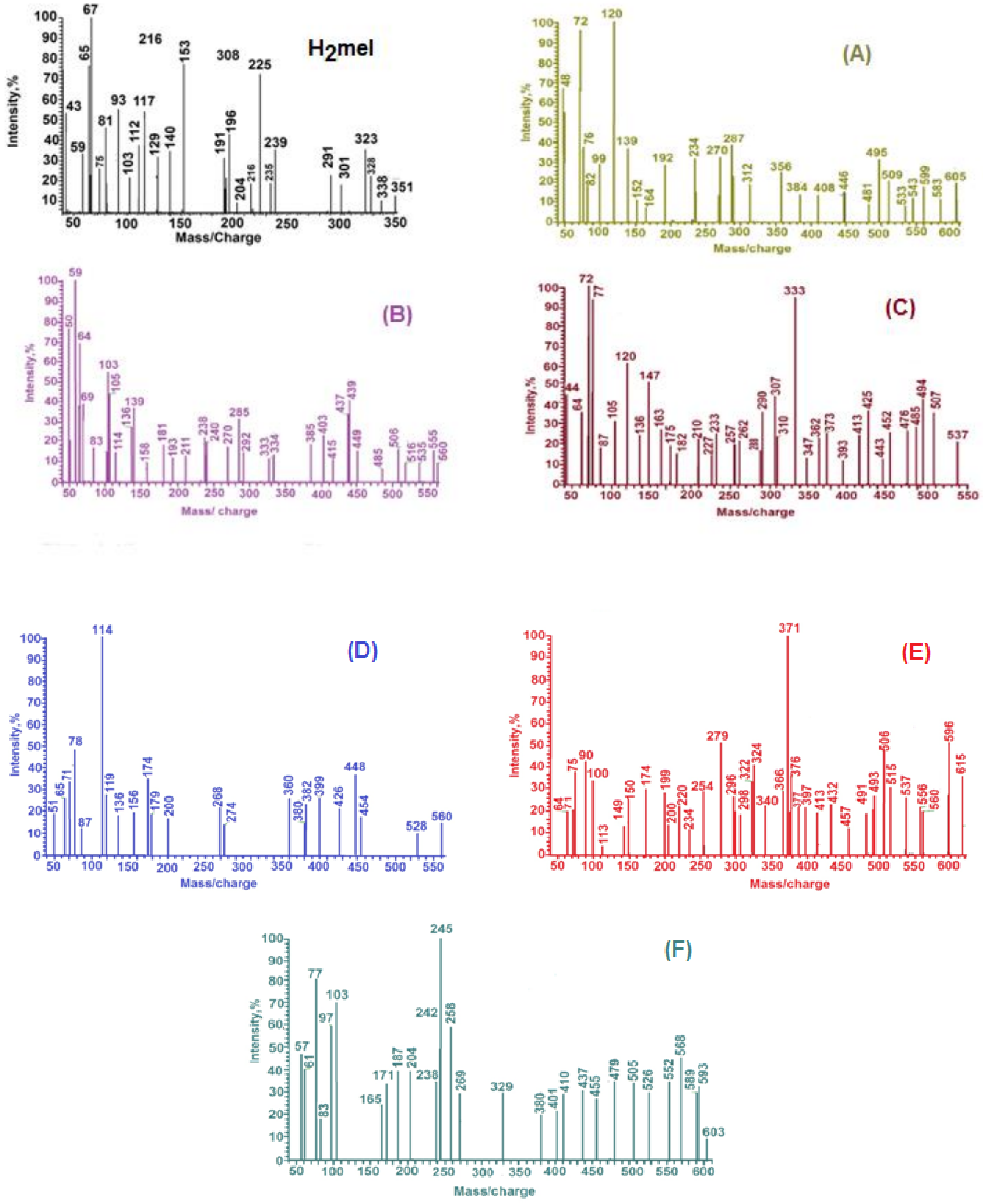

Figure S2 Mass spectra diagrams for H_2mel, Gly and their metal complexes

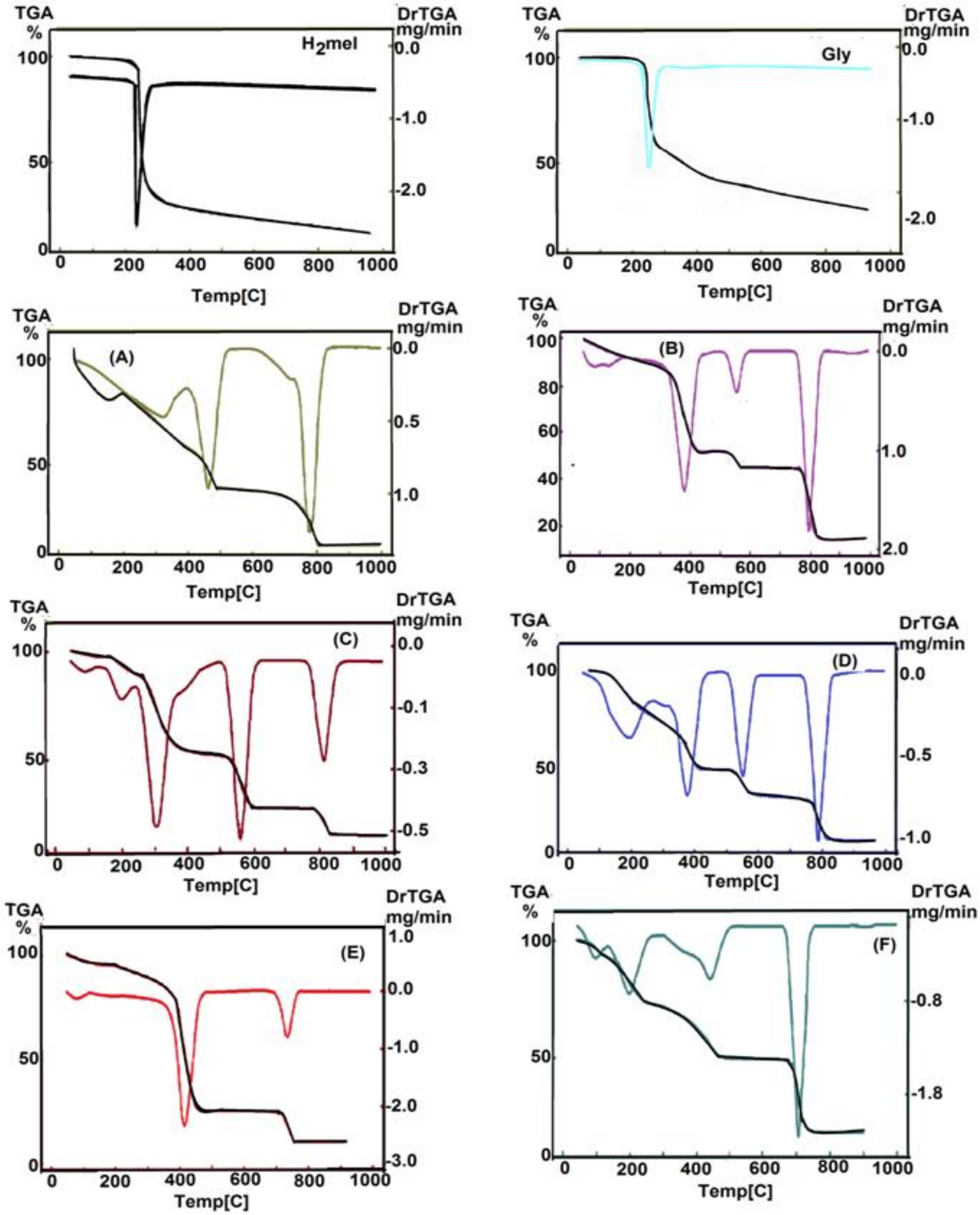

Figure 3 TG diagram forH_2mel, Gly and their metal complexes

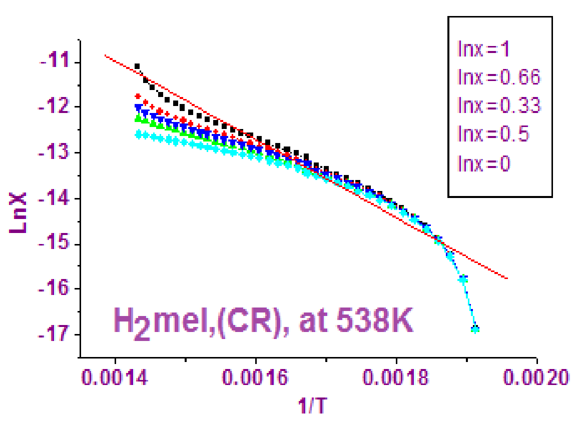
lnx=1
lnx=0.66
lnx=0.33
lnx=0.5
lnx=0
LnX
-11
-12
-13
-14
-15
-16
-17
H2mel,(CR), at 538K
0.0014
0.0016
0.0018
0.0020
1/T

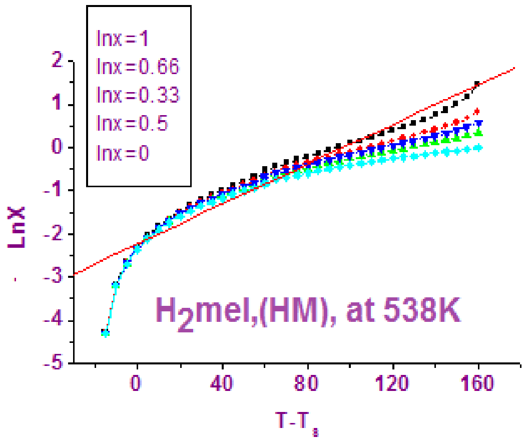
lnx=1
lnx=0.66
lnx=0.33
lnx=0.5
lnx=0
LnX
2
1
0
-1
-2
-3
-4
-5
H2mel,(HM), at 538K
0
40
80
120
160
T-Ts

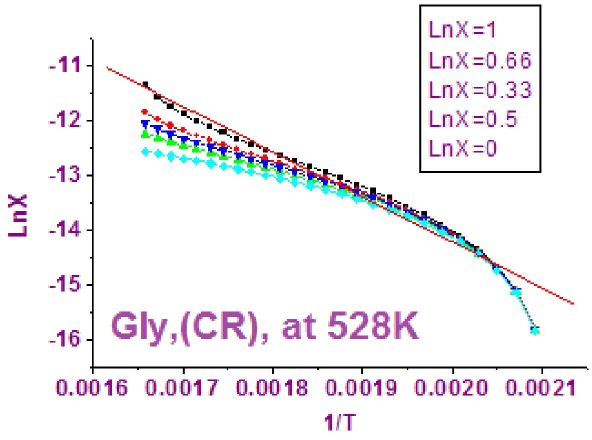
LnX=1
LnX=0.66
LnX=0.33
LnX=0.5
LnX=0
LnX
-11
-12
-13
-14
-15
-16
Gly,(CR), at 528K
0.0016
0.0017
0.0018
0.0019
0.0020
0.0021
1/T

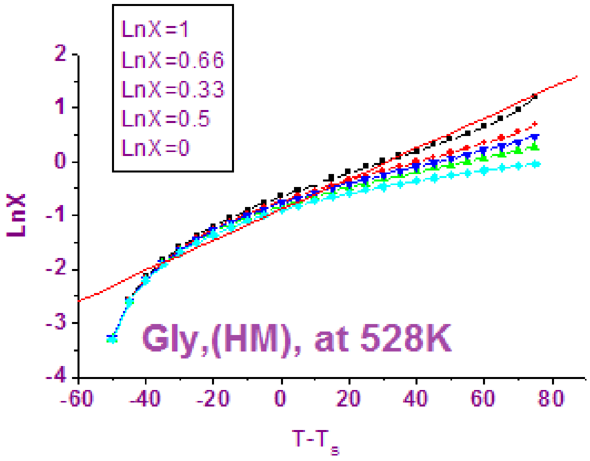
LnX=1
LnX=0.66
LnX=0.33
LnX=0.5
LnX=0
LnX
2
1
0
-1
-2
-3
-4
Gly,(HM), at 528K
-60
-40
-20
0
20
40
60
80
T-Ts

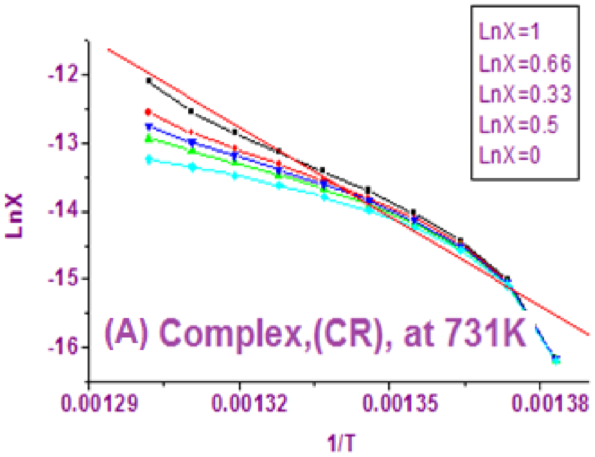
LnX=1
LnX=0.66
LnX=0.33
LnX=0.5
LnX=0
LnX
-12
-13
-14
-15
-16
(A) Complex,(CR), at 731K
0.00129
0.00132
0.00135
0.00138
1/T

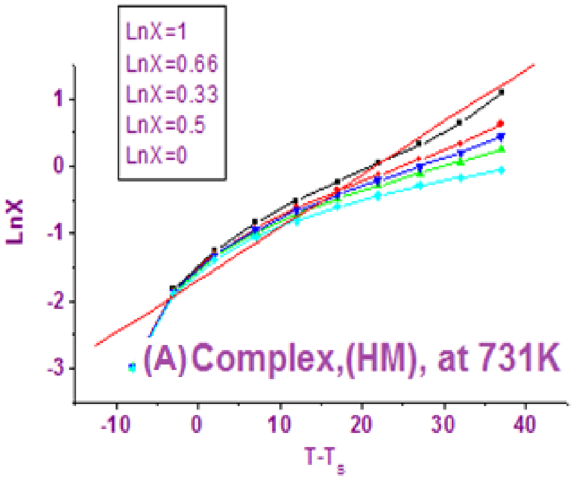
LnX=1
LnX=0.66
LnX=0.33
LnX=0.5
LnX=0
LnX
1
0
-1
-2
-3
(A) Complex,(HM), at 731K
-10
0
10
20
30
40
T-Ts

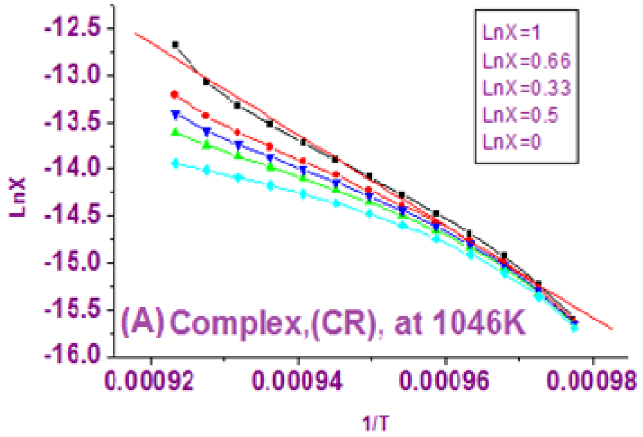
(A) Complex,(CR), at 1046K
LnX=1
LnX=0.66
LnX=0.33
LnX=0.5
LnX=0
LnX
1/T
-12.5
-13.0
-13.5
-14.0
-14.5
-15.0
-15.5
-16.0
0.00092
0.00094
0.00096
0.00098

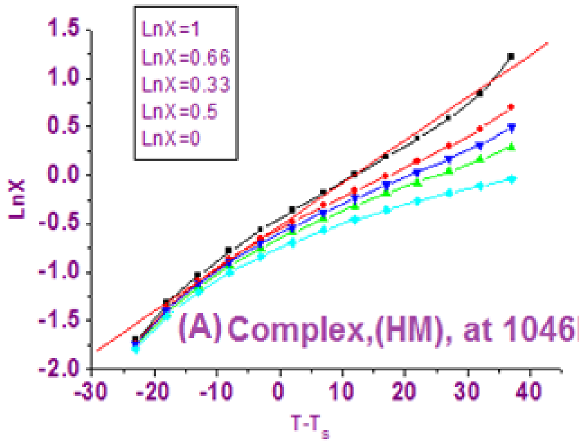
(A) Complex,(HM), at 1046K
LnX=1
LnX=0.66
LnX=0.33
LnX=0.5
LnX=0
LnX
T-Ts
1.5
1.0
0.5
0.0
-0.5
-1.0
-1.5
-2.0
-30
-20
-10
0
10
20
30
40

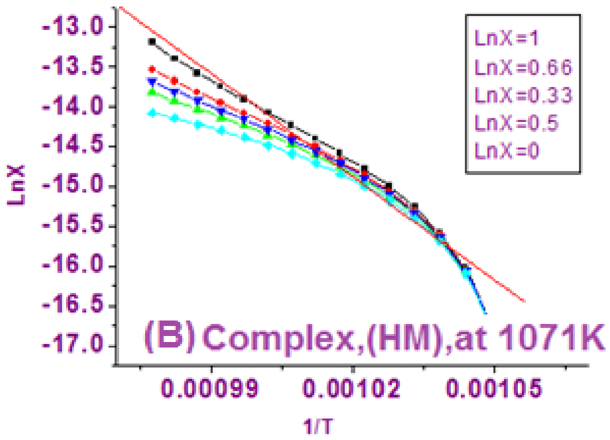
(B) Complex,(HM),at 1071K
LnX=1
LnX=0.66
LnX=0.33
LnX=0.5
LnX=0
LnX
1/T
-13.0
-13.5
-14.0
-14.5
-15.0
-15.5
-16.0
-16.5
-17.0
0.00099
0.00102
0.00105

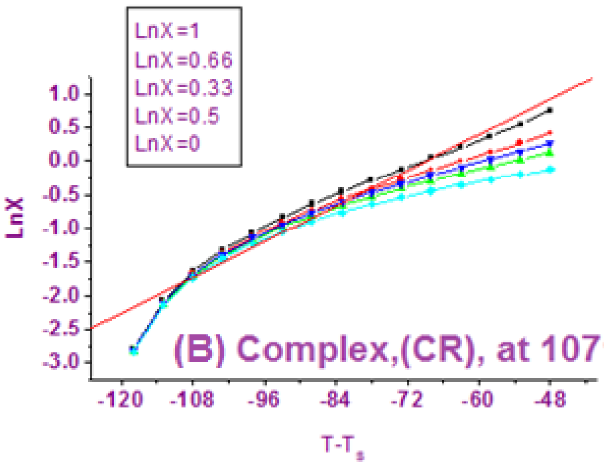
(B) Complex,(CR), at 1071K
LnX=1
LnX=0.66
LnX=0.33
LnX=0.5
LnX=0
LnX
T-Ts
1.0
0.5
0.0
-0.5
-1.0
-1.5
-2.0
-2.5
-3.0
-120
-108
-96
-84
-72
-60
-48

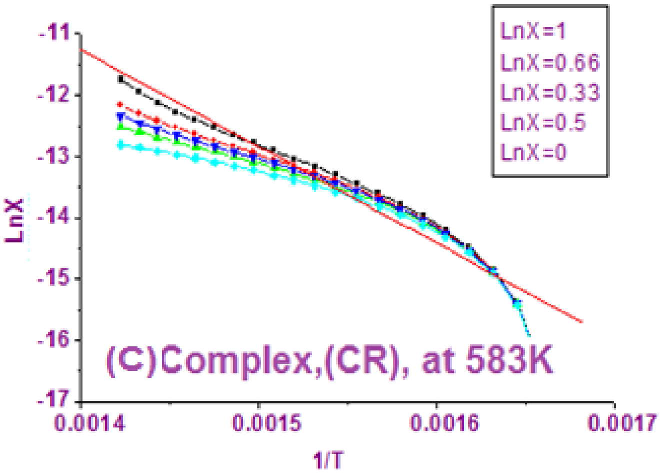
(C)Complex,(CR), at 583K
LnX=1
LnX=0.66
LnX=0.33
LnX=0.5
LnX=0
LnX
1/T
-11
-12
-13
-14
-15
-16
-17
0.0014
0.0015
0.0016
0.0017

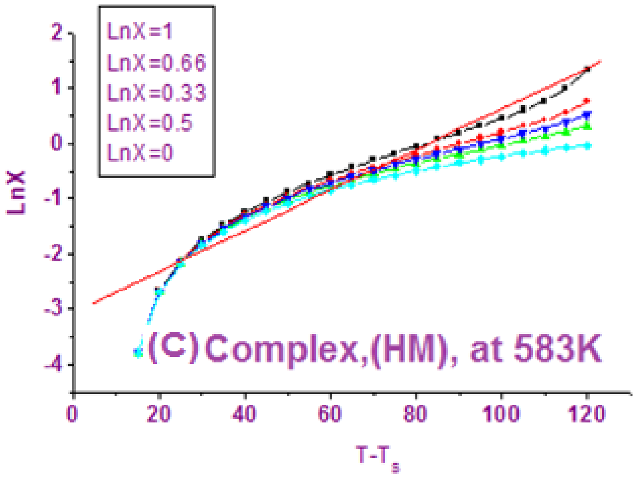
(C)Complex,(HM), at 583K
LnX=1
LnX=0.66
LnX=0.33
LnX=0.5
LnX=0
LnX
T-Ts
2
1
0
-1
-2
-3
-4
0
20
40
60
80
100
120

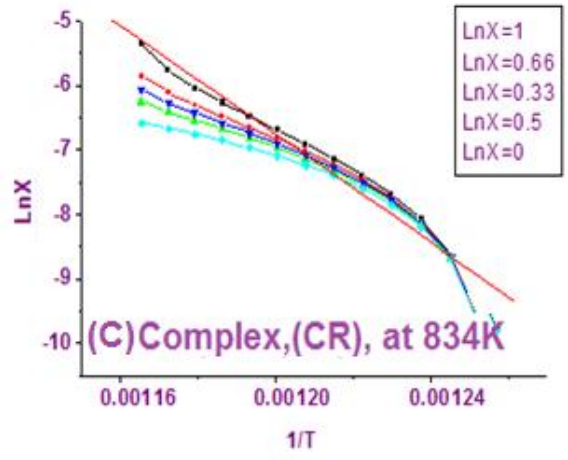
LnX=1
LnX=0.66
LnX=0.33
LnX=0.5
LnX=0
LnX
1/T
(C)Complex,(CR), at 834K

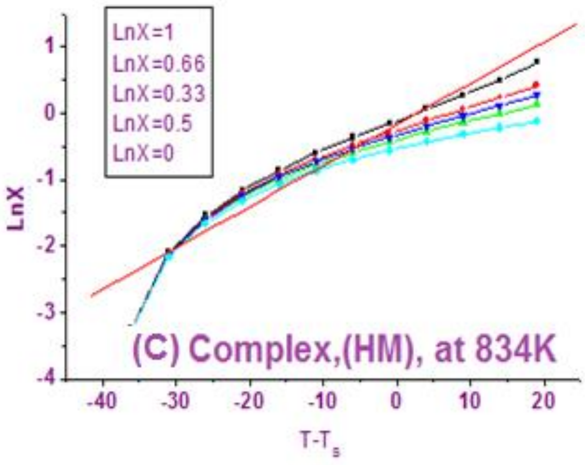
LnX=1
LnX=0.66
LnX=0.33
LnX=0.5
LnX=0
LnX
T-T_s
(C) Complex,(HM), at 834K

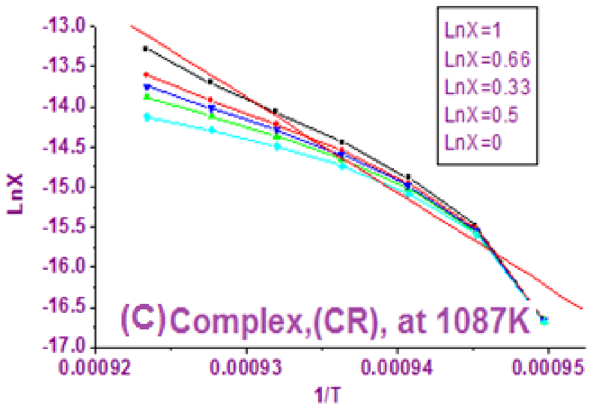
LnX=1
LnX=0.66
LnX=0.33
LnX=0.5
LnX=0
LnX
1/T
(C)Complex,(CR), at 1087K

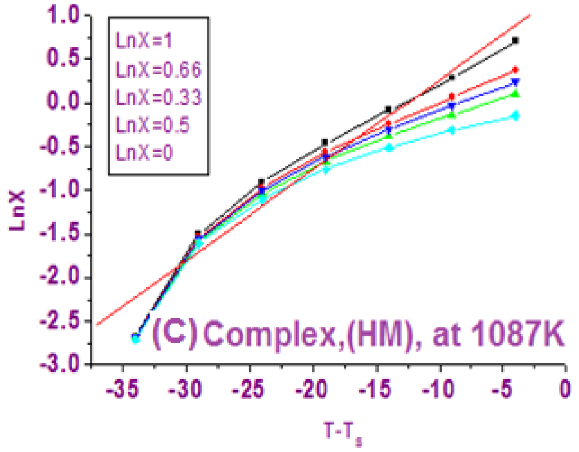
LnX=1
LnX=0.66
LnX=0.33
LnX=0.5
LnX=0
LnX
T-T_s
(C)Complex,(HM), at 1087K

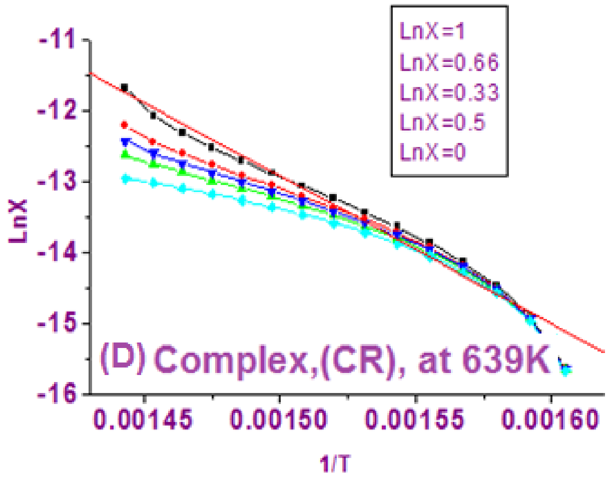
LnX=1
LnX=0.66
LnX=0.33
LnX=0.5
LnX=0
LnX
1/T
(D) Complex,(CR), at 639K

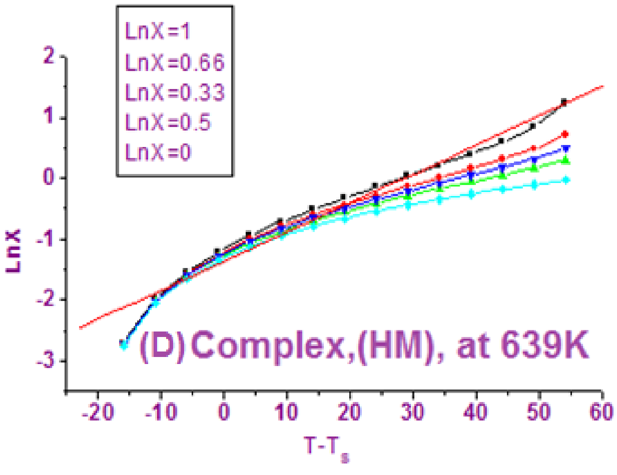
LnX=1
LnX=0.66
LnX=0.33
LnX=0.5
LnX=0
LnX
T-T_s
(D)Complex,(HM), at 639K

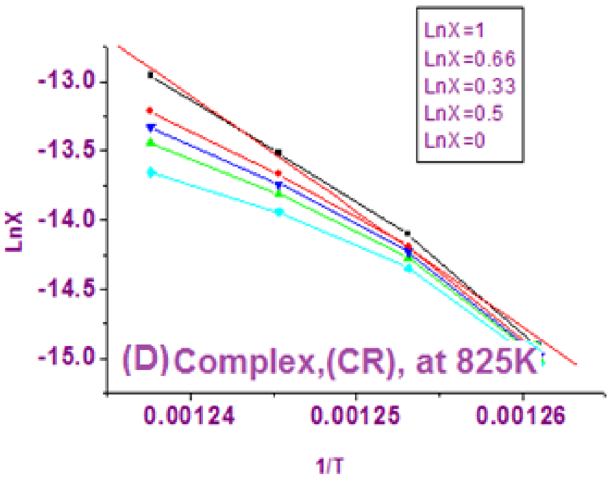
LnX=1
LnX=0.66
LnX=0.33
LnX=0.5
LnX=0
(D) Complex,(CR), at 825K
LnX
1/T

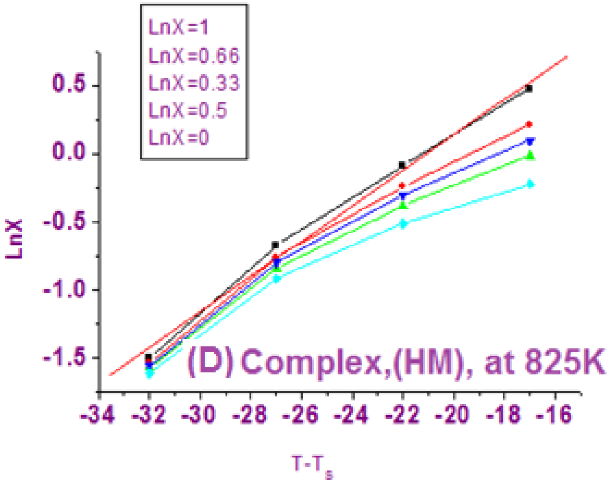
LnX=1
LnX=0.66
LnX=0.33
LnX=0.5
LnX=0
(D) Complex,(HM), at 825K
LnX
T-T_s

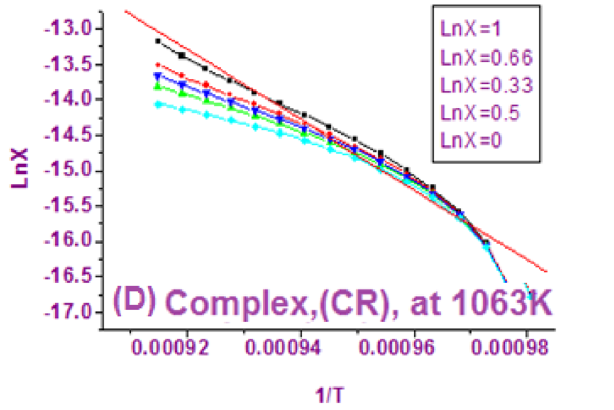
LnX=1
LnX=0.66
LnX=0.33
LnX=0.5
LnX=0
(D) Complex,(CR), at 1063K
LnX
1/T

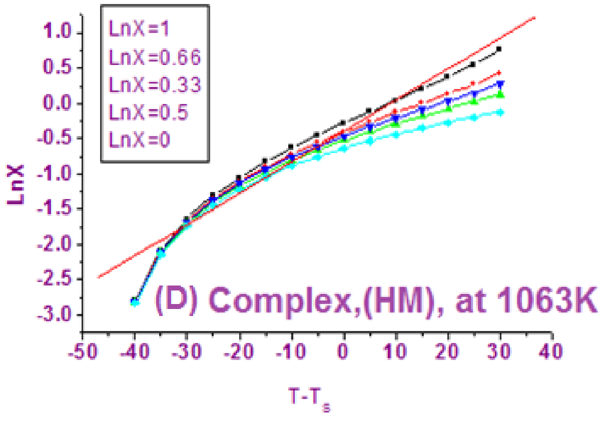
LnX=1
LnX=0.66
LnX=0.33
LnX=0.5
LnX=0
(D) Complex,(HM), at 1063K
LnX
T-T_s

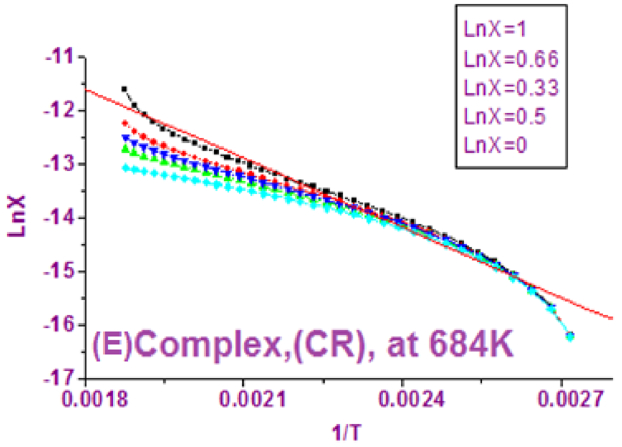
LnX=1
LnX=0.66
LnX=0.33
LnX=0.5
LnX=0
(E)Complex,(CR), at 684K
LnX
1/T

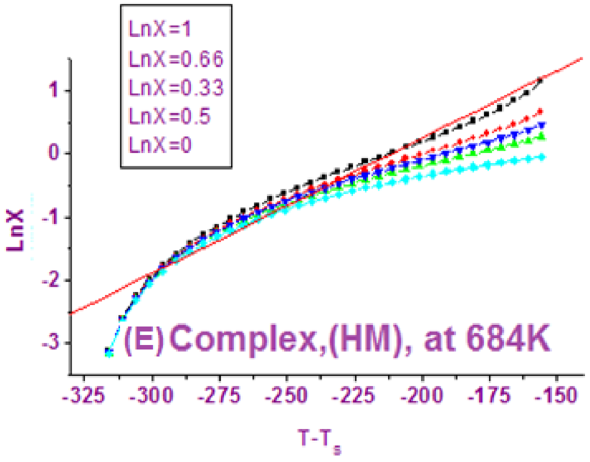
LnX=1
LnX=0.66
LnX=0.33
LnX=0.5
LnX=0
(E) Complex,(HM), at 684K
LnX
T-T_s

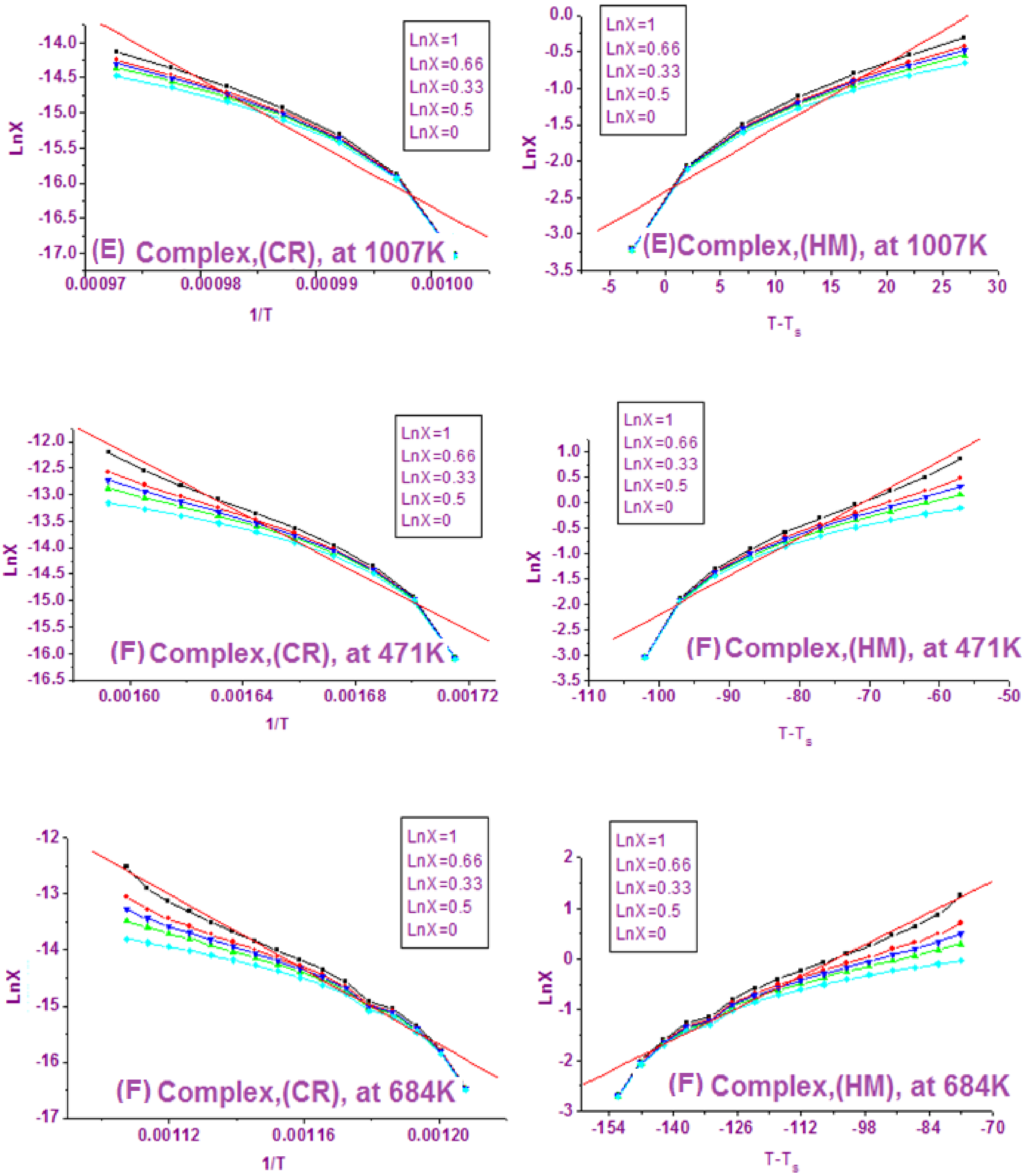

Figure S4 The diagrams of kinetic parameters of H_2mel, Gly and their metal complexes using Coats-Redfern (CR) and Horowitz-Metzger (HM) equations.

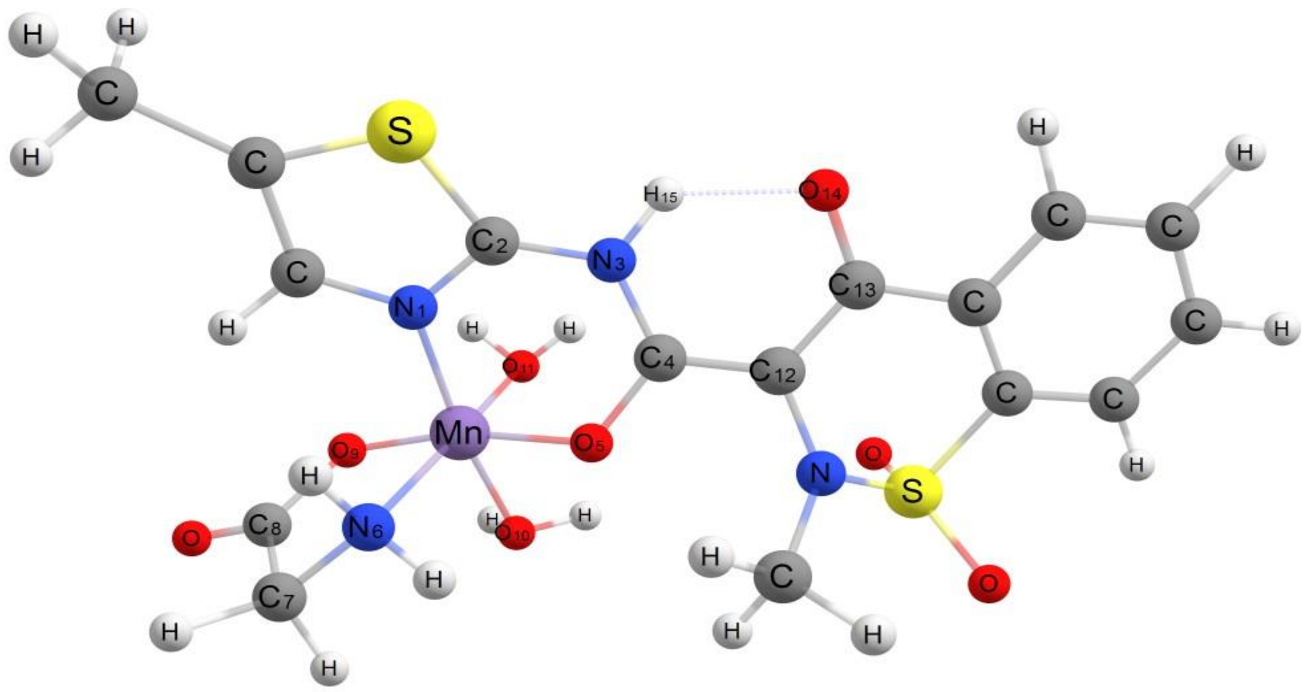

Figure S5 DFT-optimized geometry of (A) complex

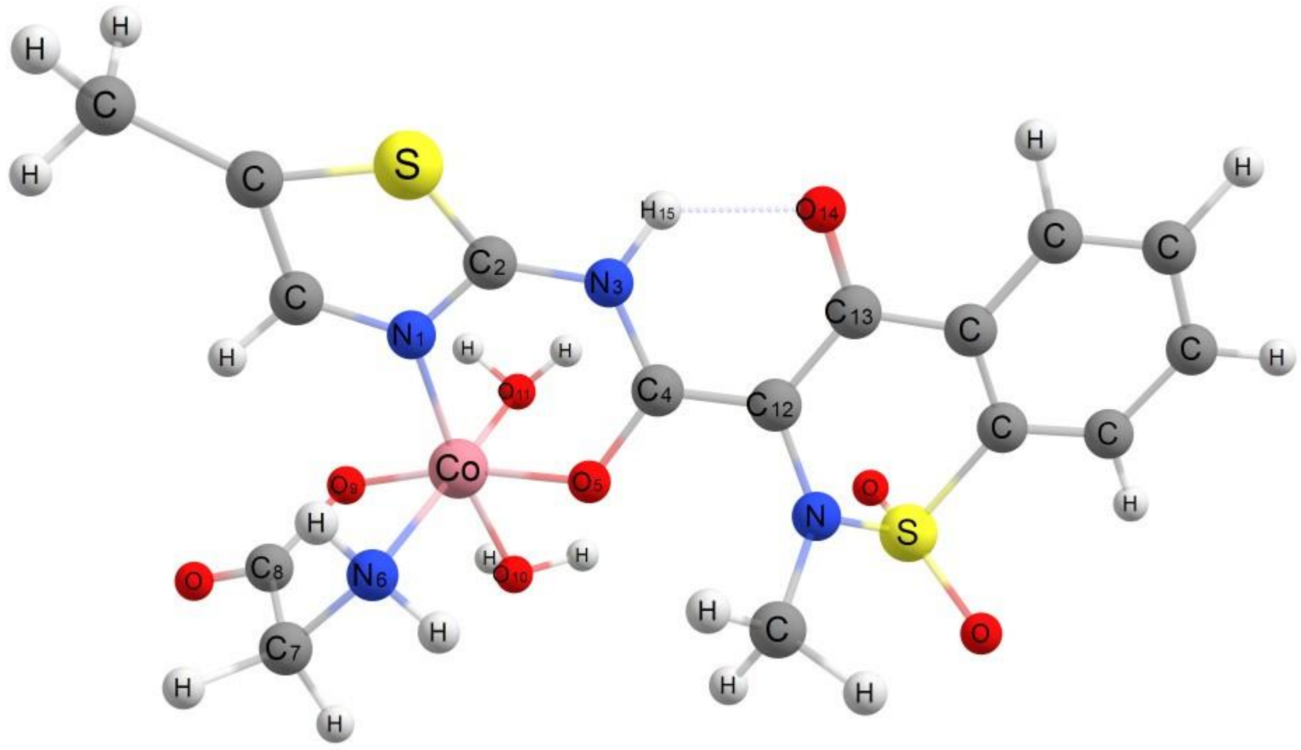

Figure S6 DFT-optimized geometry of (B) complex

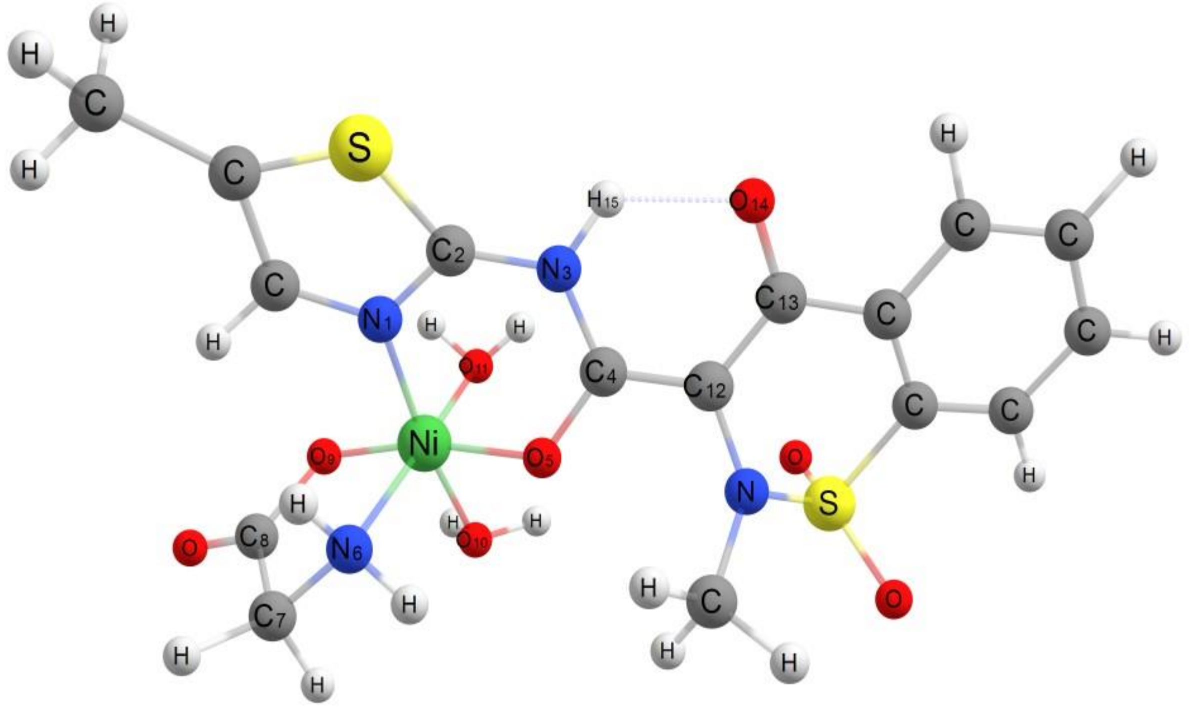

Figure S7 DFT-optimized geometry of (C) complex

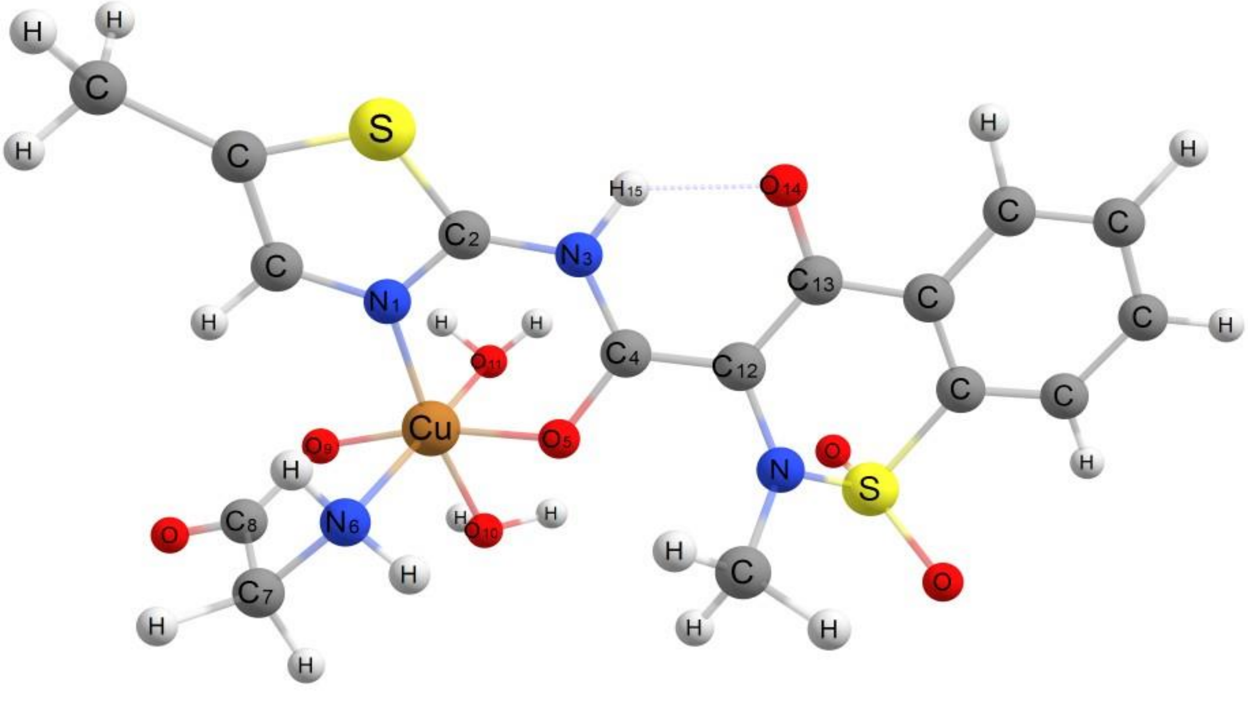

Figure S8 DFT-optimized geometry of (D) complex

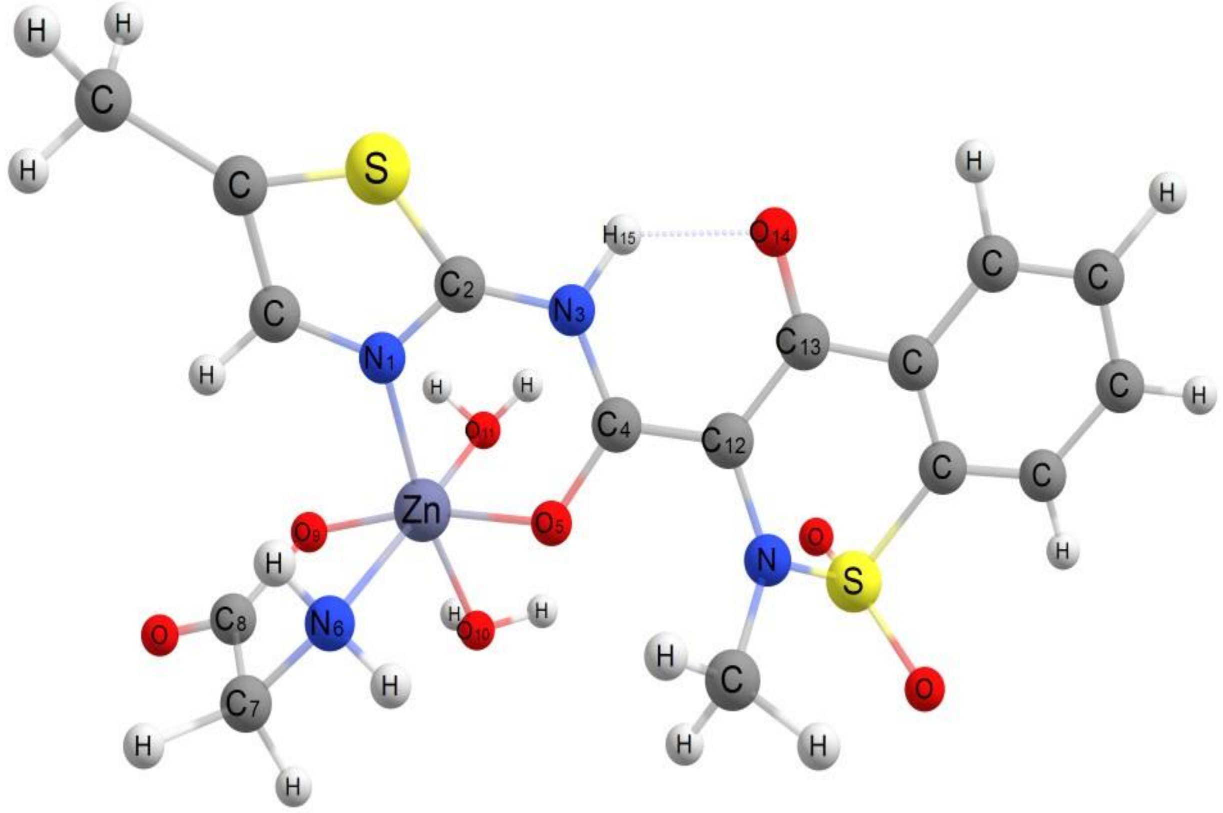

Figure S9 DFT-optimized geometry of (E) complex

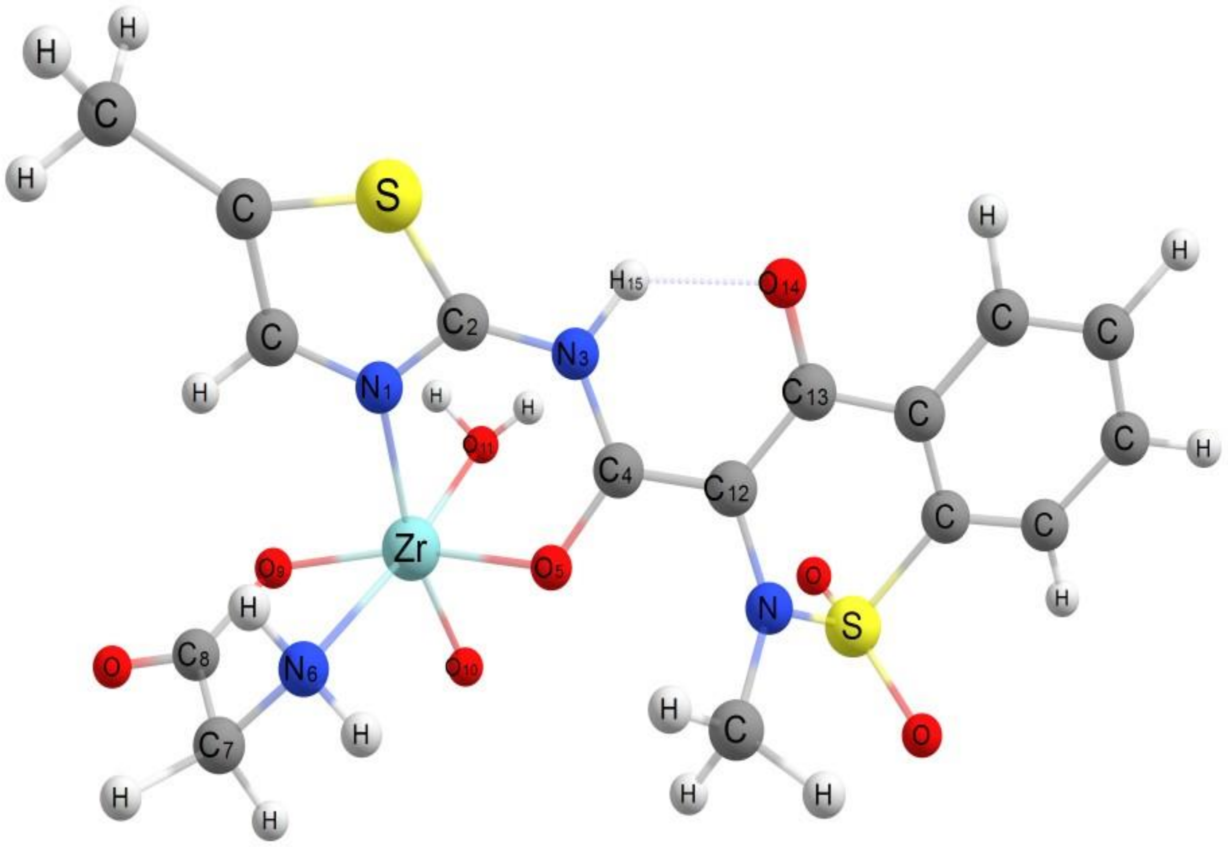

Figure S10 DFT-optimized geometry of (F) complex

Table S1 Elemental analysis and physico-analytical data for H_2mel, Gly and their metal complexes

Compounds M.Wt. (M.F.)	Yield%	Mp/°C	Color	Found (Calcd.) (%)				μ_{eff}(B.M.)	Λ Ω^{-1} mol^{-1} cm^2
				C	H	N	M		
(H_2mel)			Yellow	74.78	3.65	11.90		-	13.12
351.395 ($C_{14}H_{13}N_3O_4S_2$)				(74.82)	(3.72)	(11.97)			
	-	254					-		
Gly	-	233	White	31.87	6.56	18.55	-	-	24.20
75.067 ($C_2H_5NO_2$)				(31.94)	(6.63)	(18.61)			
(A)	86.54	270	Dark Yellow	31.61	4.85	9.15	8.97	5.62	23.50
605.392($MnC_{16}H_{30}N_4O_{13}S_2$)				(31.70)	(4.92)	(9.22)	(9.03)		
(B)	82.24	280	Orange	34.37	4.22	9.88	10.41	5.10	25.30
555.392($CoC_{16}H_{24}N_4O_{10}S_2$)				(34.52)	3.99	(10.05)	(10.59)		
(C)									
537.152 ($NiC_{16}H_{22}N_4O_9S_2$)	78.20	280	Pale Green	35.64	(4.05)	10.32	10.92	3.31	8.82
(D)				(35.71)	(4.30)	(10.42)	(10.90)	1.70	
560.008 ($CuC_{16}H_{24}N_4O_{10}S_2$)	88.26	240	Dark Green	34.18	4.18	9.79	11.24		16.82
(E)				(34.25)	(4.26)	(9.96)	(11.30)	Diamagnetic	
615.842($ZnC_{16}H_{30}N_4O_{13}S_2$)	84.24	320	Pale Yellow	30.87	4.77	8.99	10.51		26.00
(F)				(31.15)	(4.85)	(9.04)	(10.58)	Diamagnetic	
603.686($ZrC_{16}H_{24}N_4O_{11}S_2$)	89.51	310	Yellowish	31.70	3.87	9.17	15.00		11.63
				(31.78)	(3.95)	(9.25)	(15.09)		

Table S2 UV-Vis spectra for H_2mel, Gly and their metal complexes

Compounds	π-π^* transitions $\lambda_{max\ (nm)}$	H_2mel ν (cm^{-1})	ϵ Gly ($M^{-1}\,cm^{-1}$)	n-π^* transition (E) s $\lambda_{max\ (nm)}$	ν (cm^{-1}) (F)	ϵ ($M^{-1}\,cm^{-1}$)	Ligand-metal Charge transfer $\lambda_{max\ (nm)}$	ν (cm^{-1}) Assignments	ϵ ($M^{-1}\,cm^{-1}$)	d-d transition $\lambda_{max\ (nm)}$	ν (cm^{-1})	ϵ ($M^{-1}\,cm^{-1}$)
H_2mel	266	37593		362	27624		-	-	-	-	-	-
Gly	-	-		290	34482							
(A)	238<br>267<br>277	42016<br>37453<br>36101	2464<br>1275<br>1134	374	26737	1060	510	19607	250	570<br>610	17543<br>16393	125<br>120
(B)	266	37593	1007	375	26666	836	505	19801	296	605	16528	148
(C)	266	37593	1018	376	26595	924	515	19417	375	600	16666	125
(D)	265	37735	897	374	26737	430	500	20000	285	625	16000	142
(E)	236<br>266	42372<br>37593	2436<br>1088	374	26737	888	525	19047	385	-		
(F)	233<br>266	42918<br>37593	876<br>720	364	27472	235	528	18939	390	-		

Table S3 ^1H NMR values (ppm) and tentative assignments for H_2mel, Gly, (E) and (F) metal complexes

2.322-2.511	-	2.30-2.51	2.23-2.50	δH, $-CH_3$ methyline thiazolyl
	2.26-2.99	-	-	δH, $-NH_2$
2.864	-	2.75	2.86	δH, $-CH_3$ methyl
------	-	3.39-3.62	3.17-3.68	δH, H_2O
-	3.08-3.85	-	-	δH, $-CH_2$ aliphatic
7.295-7.299	-	7.01-7.62	7.08-7.14	δH, -CH thiazolyl ring
7.79-7.789	7.12-8.70	7.64-7.74	7.74-7.88	δH, -CH aromatic
8.004-8.032	-	8.01-8.03	8.05-8.96	δH, -NH amine
-	11	-	-	δH, -COOH
14.5	---	------	-------	δH, OH enolate

Table S4 Equilibrium geometric parameters bond lengths (Å), bond angles (°),dihedral angles (°), total energy (eV) , heat of formation (k cal/mol) and dipole moment of the H_2mel by using DFT calculations.

Bond length (Å)			
C7-S8	1.737	S8-O10	1.439
C1-C3	1.371	O16-H31	1.021
C1-O17	1.255	C3-N4	1.354
C1-N2	1.378	C5-O16	1.364
C3-C5	1.354	N19-C18	1.353
N4-S8	1.633	N2-H30	1.017
N4-C15	1.458	C22-N19	1.359
C18-N2	1.342	C18-S20	1.819
C21-S20	1.812	O16…..H30	1.933
S8-O9	1.439		
Bond angle (°)			
C1C3C5	118.65	N2C1O17	121.03
C1C3N4	120.15	C15N4S8	113.95
C1N2C18	125.12	N2C18S20	128.65
N2C18N19	121.59	C3C1O17	118.80
N2C1C3	119.94	C3C5O16	118.55
C6C5O16	118.88	O9S8O10	125.41
Dihedral angles (°)			
O16C5C3N4	175.29	C1N2C18S20	-8.86
O16C5C3C1	0.34	N2C1C3N4	-143.52
N19C18N2C1	166.87	C22N19C18N2	-179.99
C18N2C1C3	-179.91	N2C1C3C5	31.48
C5C3C1O17	-153.89	C18N2C1O17	5.59
C11C6C5O16	-7.71	O17C1C3N4	31.11
Total energy/ eV		-219.4285	
Heat of formation k cal/mol		-6620.3358	
Total dipole moment/D		8.57	

Table S5 Equilibrium geometric parameters bond lengths (Å), bond angles (˚), dihedral angles (˚), Total energy (eV) , Heat of formation (k cal/mol) and Dipole moment of the studied complexes by using DFT calculations.

Bond lengths/ Å	Complexes					
	(A)	(B)	(C)	(D)	(E)	(F)
M-N1	2.134	2.013	2.105	1.962	2.093	2.341
M-O5	2.091	1.908	1.985	1.931	2.067	2.187
M-N6	2.259	2.265	1.983	2.026	2.048	2.326
M-O9	2.029	2.047	1.917	1.941	2.029	2.193
M-O10	2.172	1.977	2.112	2.251	2.131	2.121
M-O11	2.191	1.986	2.133	2.258	2.137	2.123
C2-N1	1.270	1.271	1.269	1.268	1.270	1.269
C4-O5	1.361	1.359	1.360	1.358	1.362	1.361
C7-N6	1.499	1.496	1.498	1.497	1.499	1.498
C8-O9	1.349	1.348	1.347	1.346	1.349	1.351
C13-O14	1.212	1.213	1.211	1.212	1.213	1.212
O14…..H15	1.839	1.835	1.836	1.837	1.833	1.828
Bond angles/(˚)						
N1-M-O5	92.49	92.72	93.03	92.70	90.58	84.23
N1-M-N6	92.41	92.60	92.68	92.53	92.59	88.81
N1-M-O9	99.25	99.17	98.92	99.31	100.72	101.27
N1-M-O10	172.73	172.72	172.98	172.69	171.18	168.33
N1-M-O11	89.44	89.47	89.57	89.49	89.28	85.01
O5-M-N6	88.44	88.53	88.49	88.41	88.98	88.42
O5-M-O9	167.65	167.59	167.62	167.43	167.35	165.46
O5-M-O10	82.99	82.89	82.79	82.82	83.45	86.53
O5-M-O11	95.04	94.92	94.96	95.00	95.21	93.75
N6-M-O9	87.82	87.44	87.76	87.41	84.89	78.33
N6-M-O10	93.19	93.09	92.85	93.10	93.78	98.06
N6-M-O11	176.00	175.87	175.75	175.95	175.39	173.21
O9-M-O10	86.69	85.61	85.62	85.58	85.93	89.40
O9-M-O11	88.93	88.71	88.38	88.81	90.64	100.11
O10-M-O11	85.03	85.14	85.18	85.17	84.82	88.50
Total energy, eV	-330.001	-346.236	-356.422	-368.474	-317.058	-316.451
HF, k cal/mol	-8034.980	-8428.149	-8498.319	-8502.334	-8704. 269	-7824.912
Dipole moment, D	4.776	4.624	5.098	4.639	4.879	12.193
